# Cell Systems to Investigate the Impact of Polyphenols on Cardiovascular Health

**DOI:** 10.3390/nu7115462

**Published:** 2015-11-11

**Authors:** Charlotte Grootaert, Senem Kamiloglu, Esra Capanoglu, John Van Camp

**Affiliations:** 1 Laboratory of Food Chemistry and Human Nutrition, Department of Food Safety and Food Quality, Faculty of Bioscience Engineering, Ghent University, Coupure Links, Ghent 653 B-9000, Belgium; charlotte.grootaert@ugent.be (C.G.); senem.kamiloglu@ugent.be (S.K.); john.vancamp@ugent.be (J.V.C.); 2 Department of Food Engineering, Faculty of Chemical and Metallurgical Engineering, Istanbul Technical University, Maslak 34469, Istanbul, Turkey

**Keywords:** co-culture, cytokine, endothelium, intestine, liver

## Abstract

Polyphenols are a diverse group of micronutrients from plant origin that may serve as antioxidants and that contribute to human health in general. More specifically, many research groups have investigated their protective effect against cardiovascular diseases in several animal studies and human trials. Yet, because of the excessive processing of the polyphenol structure by human cells and the residing intestinal microbial community, which results in a large variability between the test subjects, the exact mechanisms of their protective effects are still under investigation. To this end, simplified cell culture systems have been used to decrease the inter-individual variability in mechanistic studies. In this review, we will discuss the different cell culture models that have been used so far for polyphenol research in the context of cardiovascular diseases. We will also review the current trends in cell culture research, including co-culture methodologies. Finally, we will discuss the potential of these advanced models to screen for cardiovascular effects of the large pool of bioactive polyphenols present in foods and their metabolites.

## 1. Introduction

The human body can be seen as an equilibrated society of human cells that communicate internally and with each other through signaling pathways including cytokines, hormones and other (circulating) structures. When food nutrients are ingested, they are converted and degraded by host and microbial enzymes, thereby generating a pool of food-derived metabolites that may affect human health. Therefore, nutrients can be seen as important circulating chemical structures that (i) can steer intestinal crosstalk with other tissues in their native form (ii) can be transported through the intestinal monolayer in an intact or modified chemical form and hence act as a “messenger molecule” to induce effects in other tissues and (iii) may have an impact on intestinal permeability for other food nutrients and/or microbial compounds towards the blood stream and may hence affect physiological processes in other organs.

In this review, we will discuss how polyphenols, a diverse group of phenolic micronutrients from plant origin, influence the inter-cellular cross-talk in the context of cardiovascular health. We will briefly discuss the gastro-intestinal fate of polyphenols in general, and then focus on different existing cell culture models and cellular assays that are generally used for mechanistic research and screening of the impact of polyphenols and their associated chemical structures on cardiovascular health. In addition, we will list possible “messenger molecules” that may be used as a biomarker to characterize these cross-talks. Finally, we will review the current trends in cell culture research and evaluate which approach is the most promising for future polyphenol research in the context of cardiovascular research.

## 2. Polyphenol Bioavailability and Bioactivity: A Complex Field of Research

### 2.1. Classification of Polyphenols and Their Dietary Sources

**Figure 1:**
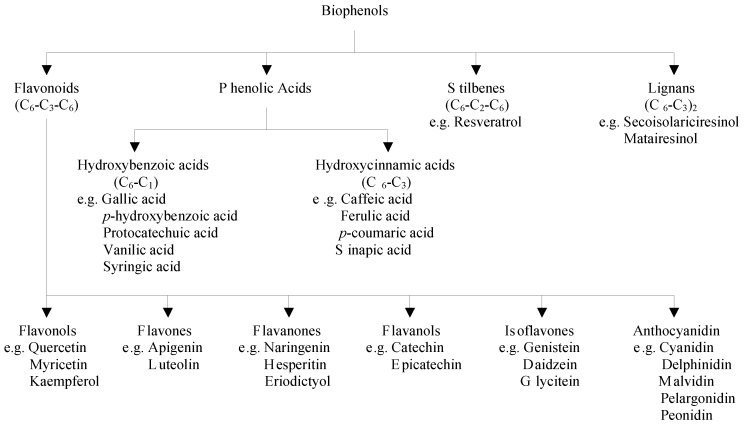
Classification of major classes of dietary biophenols.

Polyphenols are divided into several classes according to the number of phenol rings that they contain and to the structural elements that bind these rings to each other. The main groups of polyphenols are flavonoids, phenolic acids, stilbenes, and lignans [1,2] (Figure 1).

Flavonoids are low molecular weight compounds, comprising of fifteen carbon atoms, arranged in a C_6_–C_3_–C_6_ configuration. Essentially, the structure consists of two aromatic rings, joined by a 3-carbon bridge, usually in the form of a heterocyclic ring. Variations in the substitution patterns of this heterocyclic ring result in six different subclasses, namely flavonols, flavones, flavanones, flavanols, isoflavones and anthocyanidins [3]. Individual differences within each group arise from the variation in number and arrangement of the hydroxyl groups and their extent of alkylation and/or glycosylation [4,5]. Major flavonols include quercetin, myricetin and kaempferol, which are abundantly present in onions, curly kale, leeks, broccoli, and blueberries. Flavones consist mainly of glycosides of apigenin and luteolin and their important sources are parsley and celery. The main flavanone aglycones are naringenin in grapefruit, hesperetin in oranges, and eriodictyol in lemons. Flavanols exist both in monomer (catechins) and polymer (proanthocyanidins) forms. Catechin and epicatechin are the main flavanols in fruits, chocolate, red wine, and tea. Soy and its processed products are the main dietary sources of isoflavones, which contain three main molecules namely genistein, daidzein, and glycitein. The most widespread anthocyanidins are cyanidin, delphinidin, malvidin, pelargonidin, and peonidin, which are found abundantly in colored fruits including blackberry, blueberry, strawberry, and cherry [6].

Phenolic acids consist of two subgroups of hydroxybenzoic and hydroxycinnamic acids. Hydroxybenzoic acids include gallic, *p*-hydroxybenzoic, protocatechuic, vanillic, and syringic acids having C_6_–C_1_ structure, whereas hydroxycinnamic acids are aromatic compounds with a three-carbon side chain (C_6_–C_3_), with caffeic, ferulic, *p*-coumaric and sinapic acids being the most common ones [3]. Hydroxybenzoic acids are found in high concentrations in blackberry, raspberry, and tea, while blueberry and coffee are rich sources of hydroxycinnamic acids [7].

Stilbenes contain two phenyl moieties connected by a two-carbon methylene bridge [5]. The main representative of stilbenes is resveratrol, which exists in more than 70 plant species, including grapes, berries, and peanuts [2].

Lignans are produced by oxidative dimerization of two phenylpropane units. Linseed, containing secoisolariciresinol and low quantities of matairesinol, represents the main dietary source [6,7].

### 2.2. Factors Affecting the Bioavailability of Polyphenols

Many polyphenols exhibit antioxidative, anticarcinogenic, antimicrobial, antiallergic, antimutagenic, and anti-inflammatory activities [8]. In addition, the ability of polyphenols to prevent complex metabolic diseases such as obesity and diabetes has been reviewed recently [9]. The potential availability of polyphenols after digestion is important, as previous studies have stated that if the bioavailability of a certain polyphenol is poor, it would have a limited effect on health [10].

The term “bioavailability” is defined as the fraction of an ingested nutrient or compound that reaches the systemic circulation and the specific sites where it can exert its biological action [1]. Bioavailability is generally measured using *in vivo* assays (e.g., blood plasma of humans), so factors such as inter and intra-individual variability, physiological state, dose, and presence of other meal components play an important role [11]. In a critical appraisal, the main factors recognized as affecting bioavailability in humans were discussed and gathered under four main categories: factors related to the compound (chemical structure, molecular linkage, *etc.*), factors related to the food/preparation (matrix characteristics, processing, *etc.*), factors related to the host (enzyme activity, genetics, *etc.*) and external factors (food availability, different environmental factors such as climate) [12].

Polyphenols are highly diverse compounds with varying bioavailability. For instance, while soy isoflavones are well absorbed through the gut barrier, proanthocyanidins in wine and cacao are hardly absorbed. Similarly, quercetin glucosides from onions are more efficiently absorbed than quercetin glycosides such as rutin present in tea or apple [13]. Therefore it is not possible to generalize the outcomes obtained for one polyphenol to others.

### 2.3. Protective Effects of Polyphenols Against Cardiovascular Diseases

A number of epidemiological studies, clinical trials and animal experiments demonstrated that polyphenols contribute to the prevention of various degenerative diseases, including cardiovascular diseases [14]. Most of these studies have been carried out with food rich in polyphenols, in particular wine, tea, soy and berries.

Many clinical studies showed that moderate consumption of red wine is associated with reduced risk of cardiovascular diseases via reduction of oxidative stress [15,16], inflammatory biomarkers of atherosclerosis [16,17,18], improving plasma lipid markers [19], improving insulin resistance [20] and showing beneficial effects on endothelial function [21]. Animal models on mice and hamsters also confirmed the protective effect of wine against cardiovascular risk [22,23]. A strong inverse relationship between cardiovascular mortality and consumption of more than six cups of green tea per day was reported in a Japanese study [24]. In another study performed in the Netherlands, daily consumption of 3–6 cups of tea (mainly black tea) was associated with a reduced risk of cardiovascular disease mortality [25]. Moreover, in a long-term animal study both green and black teas were found to be effective in inhibiting atherosclerosis by increasing LDL/HDL ratio’s, antioxidant status and fibrinolytic mechanisms [26]. Intake of soy isoflavones was found to be protective against cardiovascular diseases by altering lipoprotein profiles in postmenopausal women [27]. Furthermore, an atheroprotective effect of soy isoflavones was also shown in mice [28]. Finally, consumption of blueberries may also have a positive effect on markers of inflammation and oxidative stress in overweight patients during childhood, and may hence protect against cardiovascular diseases associated with obesity [16].

### 2.4. Polyphenol Absorption and Biotransformation

Estimation is that only 5%–10% of the total intake of dietary polyphenols, mainly those with monomeric and dimeric structures, may be directly absorbed in the small intestine, generally after deconjugation reactions such as deglycosylation [13]. The remaining 90%–95% reach the colon where they are further metabolized to compounds with different physiological significance by the enzymatic action of the colonic bacteria [29]. In addition to metabolic changes in the gut lumen, ingested polyphenols also undergo phase I and phase II transformations in the human body. Phase I transformations consist of oxidation, reduction and hydrolysis, but these transformations occur less frequently. Phase II biotransformations taking place in the liver and the intestine occur more intensively. These phase II transformations consist of conjugation reactions where different water-soluble metabolites are formed (methyl, glucuronic and sulfate derivatives) [30]. Therefore, the polyphenols are generally present in plasma as glucuronide and sulfate derivatives.

Overall, polyphenols are known to be metabolized in the gut and the compounds initially present in the food matrix are almost absent in the tissues. Hence, the relevance of the results obtained in studies using polyphenol aglycons or specific glycosides is questionable. Chiva-Blanch and Visioli (2012), as well as Forbes-Hernandez *et al.*, (2015) question the relevance of the anti-oxidant mechanism of polyphenols, and give an overview of microbiota-related factors that are involved in the beneficial effects of polyphenols and their degradation products [31], and the molecular and cellular mechanisms related with common chronic diseases [32]. In future studies, this condition should be taken into account and the methodologies should be adopted accordingly.

## 3. Messenger Molecules in Cardiovascular Health Affected by Polyphenols

### 3.1. General Biomarkers of Cardiovascular Health

Cardiovascular diseases are the major death cause worldwide and are a class of diseases of the hearth and blood vessels, including the arteries, veins and capillaries. Diseases of the cardiovascular system include cardiac diseases, cerebrovascular diseases, and peripheral arterial diseases. Two major causes of these diseases are identified as atherosclerosis and hypertension. General prevention strategies include limited use of tobacco and alcohol, decreased psychosocial stress and, one of the most important factors: a healthy diet, more specific, sufficient consumption of vegetables and fruits, limited consumption of a (saturated) fat-rich/fiber-depleted diet, and salt reduction. To detect the onset of cardiovascular diseases, general biomarkers such as serum cholesterol level are measured in the blood plasma, yet, as cardiovascular diseases have a complex etiology, other circulating structures and cytokines can be considered as potential biomarkers. Therefore, in this review, we will focus mainly on potential biomarkers that are influenced by dietary factors. According to the PASSCLAIM consensus on criteria published by the International Life Science Institute (ILSI) in 2005, well established biomarkers for changes in the risk of cardiovascular diseases are LDL cholesterol—the hallmark for cardiovascular diseases—and blood pressure. In fact, cholesterol (LDL, HDL) is not considered as a biomarker as such, in which its presence or absence is reflecting injury or damage, but it is an abundant and natural metabolite in blood, whose quantitative variation reflects various metabolic states that are in turn reflective of cardiovascular diseases. In addition, HDL, fasting triacylglycerol and homocystein are established as examples of markers sensitive to dietary factors and are validated methodologically, but it is as yet not clear to which extent changes in these markers reflect enhanced function and reduction of disease risk. Especially for haemostatic function—which can be represented as a process in which endothelium, platelets and coagulation and fibrinolytic factors are constant in interaction with each other—and oxidative damage, there is a need to develop and validate markers of enhanced function and disease risk reduction that are sensitive to dietary changes. More and more studies also indicate that the state of low-grade inflammation, as induced by dietary factors such as a high fat diet, may influence cholesterol metabolism, and therefore C-reactive protein (CRP) and necrosis factor κB (NF-κB) have been used as potential biomarkers for cardiovascular diseases.

In the following sections, we will first discuss the role of the different organs on cardiovascular health, which cell culture models have been used to model these organs with focus on those that have been used for polyphenol research so far, and how structurally different plant polyphenols influence cell behavior and cytokine expression in these models.

### 3.2. Signal Molecules Involved in the Regulation of Cardiovascular Health

Signal molecules involved in cardiovascular health are produced by key organs such as the gastro-intestinal system, adipose tissue, endothelium, liver and immune cells. The state-of-the-art about the effects of particular polyphenols on currently existing single cell culture models representing these organs is summarized in Table 1.

**Table 1:** Impact of polyphenols on several cell line systems from intestinal, adipocyte, endothelial, liver and immune cell origin.

	Biomarkers	Polyphenols	Cell Types	Ref.
**INTESTINE**				
Transport	GLUT4, C36, FATP4	Epigallocatechin	Rat intestinal tissue	[33]
Inflammatory markers	NF-kB, TNF-α, IL-1β, IL-6	Apple peel polyphenols, Black tea polyphenols, Chrysin, Cinnamon polyphenols, Epicatechins, Epigallocatechin-3-gallate, Genistein, Grape seed polyphenols, Green tea polyphenols, Oak polyphenols, Pomegranate polyphenols, Resveratrol, Sugar cane polyphenols, Theaflavin	Caco-2/15, Caco-2, SW480, IEC6, isolated rat cells, HT-29	[34,35,36,37,38,39,40,41,42]
Cholesterol	Cholesterol uptake	Grape seed polyphenols, Red wine polyphenol, chokeberry polyphenol	Caco-2, HT29, HuTu80	[43,44]
	ApoA-1, HDL	Isoquercetin, Quercetin	Caco-2	[45]
**ADIPOSE TISSUE**				
Energy storage	Lipid staining	Blueberry polyphenols, Chlorogenic acid, Cocoa polyphenols, Ellagic acid, Epigallocatechin-3-gallate, Episesamin, Fisetin, Hydroxytyrosol, Luteolin, Maysin, Oleuropein, Resveratrol, Rutin	3T3-L1, 3T3-F442A, SGBS, hASC (human adipogenic stem cells)	[46,47,48,49,50,51,52,53,54,55,56,57]
	GLUT-4, FASN4, HSL, FAS	Daidzein, Ellagic acid, Fisetin, Hydroxytyrosol, Naringenin, Oleuropein, Pycnogenol, Resveratrol, Sakuranetin	3T3-L1, isolated human adipocytes, hASC	[46,48,57,58,59,60,61,62]
	PPAR-γ, LPL, aP2	Apple polyphenols, Catechin, Chlorogenic acid, Cocoa polyphenols, Curcumin, Cyanidin-3-*O*-glucoside, Ellagic acid, Episesamin, Fisetin, Genistein, Hydroxytyrosol, Luteolin, Maysin, Oleuropein, Protocatechuic acid, Quercetin, Resveratrol, Rutin, Sakuranetin	3T3-L1, primary human adipocytes, mesenchymal stem cells, hASC	[46,48,50,52,53,54,57,58,61,63,64,65,66,67,68,69,70,71]
	HSL, ATGL	Ellagic acid	hASC	[57]
Proliferation	MAPK, p38, Erk, JNK	Cocoa polyphenols, Curcumin, Epigallocatechin-3-gallate, Episesamin, Green tea polyphenols, Oligonol, Pycnogenol	3T3-L1, isoalted rat adipocytes, primary rat adipocytes	[52,53,62,66,72,73,74,75]
Apoptosis	caspases, PARP	Epigallocatechin-3-gallate, Episesamin	3T3-L1	[52,56]
Differentiation		Blueberry polyphenols, Curcumin, Cyanidine-3-*O*-glucoside, Delphinidin-3-*O*-glucoside, Episesamin, Genistein, Naringenin, Oleuropein, Petunidin-3-*O*-glucoside	3T3-L1, 3T3-F442A, mesenchymal stem cells	[52,55,58,68,70]
Satiety hormones	leptin, resistin, adiponectin	Apple polyphenols, Catechin, Chlorogenic acid, Cyanidin-3-*O*-glucoside, Gallic acid, Protocatechuic acid, Resveratrol, Rutin	3T3-L1, isolated human and mice adipocytes, SGBS; mesenchymal stem cells	[51,54,58,59,63,65,66,76,77,78,79,80]
Inflammatory markers	TNF-α, IL-6, IL-1β	Chlorogenic acid, Naringenin, Oligonol, Quercetin, Resveratrol, Rutin	3T3-L1, 3T3-L1/RAW263 coculture, isolated human and rat adipocytes, human primary adipocytes	[52,54,67,74,81,82,83]
	MCP-1	Naringenin, Quercetin, Resveratrol	primary human adipocytes, 3T3-L1/RAW263 coculture	[67,81]
Hypoxia	VEGF	Cinnamon polyphenols, Episesamin, Resveratrol,	3T3-L1, isolated adipose tissue	[52,82,83]
	C/EBPα	Ellagic acid	hASC	[57]
**ENDOTHELIUM**				
Transport	GLUT-4, Akt	Silibinin, Xanthohumol	HUVEC	[84]
Vasorelaxation	NO, eNOS	Red wine polyphenols, Resveratrol, Sinapic acid	EaHy.926, HUVEC	[85,86,87]
	ACE	Billberry anthocyanidins, Butein, Kaempferol Oak polyphenols, Tannins, Tea polyphenols	ACE-test, HUVEC	[88,89,90,91,92,93]
	ET-1	Quercetin	Isolated human umbilial chord veins	[94]
Proliferation	MAPK, p38, Erk, JNK	Apigenin, Catechins, Cocoa procyanidins, Genistein, Quercetin,	EC, VSMC, HMEC, HUVEC	[95,96,97]
Migration	MMPs	Cyanidin, Delphinidin, Epigallocatechin-3-gallate, Green tea polyphenols, Hydroxytyrosol, Isoxanthohumol, Malvidin, Oleuropein, Pelargonidin, Peonidin, Petunidin, Quercetin, Resveratrol, Xanthohumol	HUVEC, HMEC-1	[84,95,98,99]
Tubulus formation		Hydroxytyrosol, Oleuropein, Quercetin, Resveratrol Xanthohumol,	HUVEC and HMEC-1	[95,98,100]
Inflammatory markers	NF-κB, TNF-α	Catechins, Isoxanthohumol, Silibinin	HUVEC, VSMC	[84,95,96]
	COX-2	Hydroxytyrosol, Oleuropein, Quercetin, Resveratrol	EC	[98,101]
**LIVER**				
Energy metabolism	Ser9 and Ser641 glycogen synthase	Epigallocatechin	HepG2, isolated rat hepatocytes	[102,103]
	fat storage	3-caffeoyl,4-dihydrocaffeoylquinic acid, Blueberry anthocyanins, Curcumin, Cyanidin-3- glucoside, Ellagic acid, Ginko bilonba polyphenols, Quercetin, Resveratrol, Sechium edule shoots polyphenols	HepG2, H4IIEC3, Huh7, isolated rat hepatocytes	[57,104,105,106,107,108,109,110,111,112,113,114,115]
	CPT-1, ACC	Cyanidin-3-*O*-β-glucoside, Ginko biloba polyphenols, Resveratrol, Sechium edule shoots polyphenols	isolated rat hepatocytes, HepG2	[107,110,111,116,117,118]
	AMPK, LXR, FAS, PPAR-α, SREBP1c	3-caffeoyl,4-dihydrocaffeoylquinic acid, Blackberry polyphenols, Cocoa polyphenols, Curcumin, Cyanidin-3-*O*-β-glucoside, Cyanidin chloride, Ellagic acid, Epicatechin, Epigallocatechin-3-gallate, Ginko biloba polyphenols, Mulberry anthocyanins, Resveratrol, Sechium edule shoots polyphenols, Sweet potato anthocyanins	HepG2, isolated rat hepatocytes, Huh7	[57,103,104,107,109,110,111,112,113,116,117,118,119,120,121,122,123,124,125]
	Akt/PI3K	Epicatechin, Quercetin	HepG2	[126,127]
	GPAT1	Cyanidin-3-*O*-glucoside	HepG2	[115,117]
Cholesterol metabolism	Cholesterol storage	Grape seed polyphenols, Red wine polyphenols	HepG2	[43]
	ApoA1, ApoB100, HDL, HMGCoR	Epigallocatechin, Epigallocatechin gallate, Gallic acid, Quercetin, Red wine polyphenols, Resveratrol, Sechium edule shoots polyphenols	HepG2	[45,107,128,129]
Apoptosis	others (DNA fragmentation, PI staining)	Cyanidin-3-ol	HepG2	[130]
	Caspases	Black tea polyphenols, Epigallocatechin-3-gallate, Quercetin, Resveratrol, Solanum nigrum polyphenols	HepG2, HLE	[106,126,131,132,133]
**IMMUNE CELLS**				
Inflammatory markers	MCP-1, NF-κB, COX-2; TNF-α; IκBα; IL-1α; IL-1β; IL-6; IL-8; IL-10	Cacao polyphenols, Caffeic acid, Caffeoylquinic acids, Curcumin, Cyanidin-3-*O*-β-glucoside, Epicatechin, Gallic acid, Grape seed proanthocyanidins, Hydroxytyrosol, Naringenin chalcone, Oleuropein, Olive oil polyphenols, Quince peel polyphenols, Resveratrol, Rosmarinic acid	THP-1, RAW 264.7, HMC-1, NR8383, U-937	[81,134,135,136,137,138,139,140,141,142,143,144,145,146,147]
Proliferation	MAPK, p38, ERK1/2	Quince peel polyphenols, Resveratrol	THP-1, HMC-1	[135,138,147]
Vasorelaxation	eNOS, NO	Cacao polyphenols, Epicatechin, Hydroxytyrosol, Naringenin chalcone, Resveratrol	THP-1, RAW 264.7	[81,134,135,140]
Apoptosis	PI3K, Akt	Quince peel polyphenols, Resveratrol	THP-1	[135,147]
Migration	MMPs	Olive oil polyphenols	THP-1	[141]
Energy metabolism	PPAR-γ; LXR-α	Cyanidin-3-*O*-β-glucoside	THP-1	[136]

#### 3.2.1. The Gastro-Intestinal Tract

The gastro-intestinal tract is a complex system consisting of stomach, small intestine and large intestine. At cellular level, strong local differences exist in morphology and function of the digestive cells. The gut epithelium consists of (i) enterocytes involved in the production of digestive enzymes and the active transport of food compounds; (ii) mucin secreting goblet cells; (iii) entero-endocrine cells involved in the production of gut hormones involved in hunger and satiety; and (iv) different types of cells involved in immune response regulation (such as M-cells of follicle-associated epithelium in Peyers patches), and transport of antigens (such as dendritic cells which are in contact with T-cells from mesenteric lymphoid nodes) [148]. Therefore, the gut epithelium can be considered as a key organ in the inter-organ cross-talk in metabolic homeostasis for the following reasons: (i) it is the first barrier that nutrients have to cross before entering into the blood stream; (ii) it is involved in hormones that have an impact on energy intake, and (iii) it is considered as the main immunity-regulating organ in the body as 70% of the immune cells are located in the gastro-intestinal tract. The gut cells contain transporters for glucose (glucose transporter 4 (GLUT4)) and fat (cluster of differentiation (CD36), fatty acid transporter protein 4 (FATP4)), as well as enzymes involved in lipoprotein assembly (microsomal triglyceride transfer protein (MTP)). In addition, they secrete cytokines involved in inflammatory processes such as tumor necrosis factor α (TNF-α), interleukin-1β (IL-1β) and interleukin-6 (IL-6), and use the NF-κB pathway to regulate inflammation and proliferation.

Caco-2 cells are probably the most studied cell culture to investigate the transport kinetics of food compounds by an intestinal monolayer. The Caco-2 model is a continuous cell line that is derived from a colon adenocarcinoma, and has the useful property that it spontaneously differentiates upon confluency towards a polarized epithelium of well-developed enterocyte-like cells with microvilli. Differentiation markers also include the development of tight junctions, and the expression of specific digestive hydrolases such as alkaline phosphatase, aminopeptidase N and A, sucrase, isomaltase, dipeptidyl peptidase IV, and endopeptidase. Transport proteins, enzyme receptors, ion channels, and lipid molecules are also situated on the apical part [149,150,151]. The housekeeping functions, which are responsible for the maintenance of the intra- and extracellular environment, are situated in the basolateral part of the cells [152]. The HT-29 cell line is a cell line from colorectal origin with epithelial morphology, and has been used as a model for absorption, secretion and transport by intestinal cells. Under standard culture conditions, these cells grow as a non-polarized, undifferentiated monolayer. Yet, altering culture conditions or treating the cells with different inducers, results in a differentiated and polarized morphology, characterized by a redistribution of membrane antigens and development of an apical brush-border membrane [153]. Other human intestinal cell lines are less popular for the simulation of the human intestinal epithelium, such as the SW480 cell line, which is mainly used in unravelling cancer-related mechanisms, and the HuTu80 cell line, a model for duodenal cells.

The Caco-2 cell line has extensively been used to model transport of dozens of structurally different polyphenols [154]. One major characteristic is the cells potential to perform phase II transformations including glucuronidation, sulfation, and acetylation of the aglycon polyphenol. Intestinal cell lines have been used to investigate the impact of polyphenols on glucose and lipid transporters, cholesterol metabolism, and immune response. Epigallocatechin had a strong influence on nutrient transporters. Wine polyphenols as well as quercitin and isoquercitin have been shown to modulate cholesterol metabolism, whereas a wider range of polyphenols was able to modulate inflammatory markers (Table 1).

#### 3.2.2. The Adipose Tissue

In the past, the adipose tissue was considered as a fat depot tissue spread throughout the body, storing the left-overs of the energy that were taken in, and releasing energy when required. Nowadays, the adipose tissue is widely recognized as a fully operational organ, with a complex endocrine signaling potential that not only regulates fat storage and release, but also satiety and even immune response and angiogenesis. Lipid metabolism is directly related to circulating cholesterol, triglyceride and fatty acid levels, and therefore the fat tissue, as well as the liver, are considered as significant players in cardiovascular diseases. The size of the fat tissue is determined by the size of the fat cells and the cell number. The adipocyte number is the result of the balance between cell proliferation (mediated by mitogen-activated protein kinase (MAPK) pathways), apoptosis (regulated by caspases, poly ADP ribose proliferator (PARP)), and differentiation (marked by the expression of lipid and glucose uptake enzymes, as well as secretion of adipokines). Insulin, secreted by the β-cells in the pancreas, is the major hormone controlling glucose and lipid metabolism. It activates the insulin receptor tyrosine kinase (IR), and results in recruitment of insulin receptor substrates (IRS-1), which can also be activated upon binding of the growth factor insulin growth factor-1 (IGF-1) to its receptor. IRS-1 is transmitting the signal to three intracellular pathways of the MAPK family, more specific extracellular signal regulated kinase (ERK), p38 kinase and c-Jun N-terminal kinase (JNK). Activation of these pathways results in adipocyte proliferation and protein synthesis. IRS-1 is also transmitting the signal to Akt, the key molecule in insulin sensitive tissues, via activation of phosphatidylinositol 3-kinase (PI3K). Akt stimulation results in glucose uptake through GLUT 1 and GLUT4, decreased lipolysis and increased lipogenesis [155]. Adipocytes store energy originating from triglycerides, fatty acids and glucose, which is converted to triglycerides through *de novo* lipogenesis. Briefly, glucose is taken up into the adipocyte through insulin-mediated GLUT4, converted to pyruvate, and transported into the mitochondria where it is converted to malonyl CoA. Cytosolic fatty acid synthase (FASN) is involved in the stepwise elongation of malonyl CoA to fatty acids. In a state of negative energy balance, adipocytes release fatty acids to provide energy to the peripheral tissues (lipolysis). Lipases such as hormone-sensitive lipase (HSL) play a major role in this process. Also peroxisome proliferator-activated receptor γ (PPARγ), a nuclear transcription factor that induces lipoprotein lipase (LPL) and adipocyte protein 2 (aP2), is strongly involved in fatty acid storage and glucose metabolism [156]. Fully differentiated adipocytes express leptin, adiponectin and resistin, which are hormones with a major impact on hunger and satiety. In addition, adiponectin [157,158], resistin [159], and apelin [160] have been considered the key molecules that make the link between the “twin epidemics” obesity and diabetes, and are also involved in the pathology of cardiovascular diseases [161].

Finally, a chronic low-grade inflammation of the adipose tissue may also contribute to the development of cardiovascular diseases [162]. Inflammation generally results in increased insulin resistance, as well as in macrophage infiltration mediated by macrophage attraction factors (MCP-1). The low-grade inflammatory tone (marked by increased TNF-α, IL-1β and IL-6 secretion) has been partially attributed to increased circulating lipopolysaccharide (LPS) levels, which is the result of enhanced permeability of the intestine for microbial compounds, and to hypoxic conditions generated by the increased size of the adipocytes. Hypoxic conditions result in the expression of vascular endothelial growth factor (VEGF), which is one of the key molecules triggering the angiogenesis process. Collaboration between the fat cells and vascular system is indispensable to develop a fully functional adipose tissue.

Adipose tissue research has mostly been performed with isolated primary adipocytes, or immortalized murine 3T3-L1 cells due to the lack of a continuous human white adipose tissue cell line. Human cell lines that have been used to investigate adipocyte differentiation are the Simpson-Golabi-Behmel Syndrome (SGBS) cell line [163], the brown adipocyte cell line PAZ6 [164], the TAH9 cell line derived from white adipose tissue but with low differentiation potential [165] and human liposarcoma cell lines (LiSa-2, LS 14, LS857 and LS707) [166,167]. Recently, primary adipocytes became commercially available through the American Type Culture Collection (ATCC). Therefore, nowadays, more options are available to investigate human adipocytes.

A wide set of structurally different polyphenols are affecting pathways involved in energy storage, proliferation as well as apoptosis, differentiation, satiety hormones, inflammatory markers, and hypoxia. In particular, polyphenols present in grapes (e.g., resveratrol), vegetable oils (e.g., oleuropein, hydroxytyrosol, episesamin), tea (e.g., epigallocatechin) and berries (e.g., anthocyanins) are shown to play an effective role in inhibiting adipogenesis and cell proliferation (Table 1). These results demonstrated that several polyphenols might be evaluated as novel potential complementary treatments for associated cardiovascular diseases.

#### 3.2.3. The Endothelium

The endothelium is a thin layer of cells that lines the interior surface of blood vessels and lymphatic vessels. Vascular endothelial cells line the entire circulatory system, and have distinct functions including (i) a barrier function; (ii) blood clotting; (iii) hormone trafficking; (iv) inflammation regulation; (v) angiogenesis; and (vi) vasoconstriction and -dilatation.

Endothelial cells are a selective barrier (mediated by junction proteins such as vascular endothelial cadherin (VE-cadherin)) that contain fatty acids and glucose transporters (GLUT4, CD36) for the transport of nutrients, the latter are activated through protein kinase B (Akt). Endothelial dysfunction is a key event in the early stage of atherosclerosis and is often found in patients with coronary heart disease, type II diabetes, hypertension and hypercholesterolemia. Endothelial dysfunction is marked by increased reactive oxygen species (ROS) production and decreased nitric oxide (NO) production (result of activation of sirtuins (SIRT) and reduction of endothelial nitric oxide synthase (eNOS)). NO is involved in vasorelaxation, and may hence be a target to treat hypertension. Other molecular targets for vasodilatation are (i) proteins involved in the rennin-angiotensin-aldosterone system, such as angiotensin converting enzyme (ACE) and its receptors, that are involved in the regulation of blood pressure and water balances and (ii) endothelins (ET-1), which are proteins upregulated in response to hypoxia, oxidized LDL, pro-inflammatory cytokines, and bacterial toxins, and have an impact on blood pressure [168].

Angiogenesis is the process of new blood vessel formation characterized by an increased proliferation, migration, differentiation, and tubule formation of the endothelial cells. Proliferation is regulated by the MAPK-pathway. Matrix metalloproteinases (MMPs) are involved in the degradation of the extracellular matrix to allow endothelial cells to migrate in the tissue. VEGF, produced by the adipose tissue, is the main attraction factor for endothelial cell migration.

The low-grade inflammatory tone in metabolic syndrome patients has an impact on the endothelium, which is characterized by increased expression of transcription factors (NF-κB), enzymes (cyclo-oxygenase 2 (COX-2)) and cytokines (TNF-α, ICAM-1, MCP-1) involved in the inflammation process.

Unlike other cell types, for the endothelial cells, the increase in extracellular glucose levels is not accompanied by a decrease in the rate of transmembrane transport. This property makes the endothelial cells very sensitive towards hyperglycemia induced dysfunction, such as irreversible arterial stiffness caused by continuous glucose exposure [169].

Until now, most studies used primary HUVEC cells as a model for human endothelium. The HUV-EC-C cell line is similar to primary HUVEC cell lines, but these cells can be cultivated until 50 to 60 doubling times, which is at least ten times more than primary HUVEC cells. These cells are widely used to investigate the effect of drugs and nutrients on wound healing, angiogenesis (consisting of cell migration, differentiation and tubule formation), the production of NO, ROS, and monocyte/leukocyte attraction factors such as vascular cell adhesion molecule 1 (VCAM-1) and ICAM-1 and the expression and activity of ACE. Endothelial cells can be investigated as such, or challenged with cytokines such as TNF-α, IL-1β, and VEGF to mimic the inflammatory and hypoxic signaling in cardiovascular disease.

Other cell types that have been used to study vascular effects are primary microvascular retinal endothelial cells (HMREC), and immortalized human microvascular endothelial cells (HMEC-1) [170]. The HMEC-1 cell line has a cobblestone morphology when grown in monolayer culture, expresses and secretes von Willebrand's Factor involved in coagulation, take up acetylated low-density lipoprotein, and rapidly form tubes when cultured on matrigel. They express cell-surface molecules typically associated with endothelial cells, including CD31 and CD36, and cell adhesion molecules such as ICAM-1 and CD44.

The ISO-HAS cell line has been established from tumor tissue of a human hemangiosarcoma, and has a life span of more than 100 passages [171]. This cell line has a cobble-stone morphology at confluency, contact-inhibited growth, active uptake of acetylated LDL and CD31 expression. Yet, because of the lack of the von Willebrand factor and tube-formation activity, as well as their high tumor-forming capacity in mice indicate that this is a malignant and poorly differentiated cell line. The EA.hy926 is a human somatic cell hybrid continuous cell line with endothelial properties. Compared to HMEC cells, this cell line is less capable of tube formation and has significant differences in expression profiles [172]. The EA.hy926 cell line has been used to investigate hyperglycemia induced stiffness and blood pressure mechanisms [87,169].

These cell systems have been used to investigate the impact of a wide variety of polyphenols on nutrient transport, vasorelaxation, cell proliferation, tubulus formation, and inflammatory responses. Plant polyphenols including tea, cacao and bilberry polyphenols are shown to inhibit angiogenesis through regulation of multiple signaling pathways. Moreover, studies pointed out that resveratrol stimulate nitric oxide production, whereas tannins and sinapic acid exert antihypertensive effects (Table 1). Overall, studies indicated polyphenols as modulators of endothelium through different mechanisms.

#### 3.2.4. The Liver

The liver is a gland, which is composed of liver lobule units containing 80% (*v*/*v*) functional parenchymal cells, also called hepatocytes. The other non-parenchymal cells consist of hepatic stellate cells, sinusoidal endothelial cells, and phagocytic cells. The liver has a profound impact on energy metabolism, as it contains the major stock of glycogen in our body which is mediated through glycogen synthases, and is strongly involved in lipid metabolism by regulating lipogenesis as well as fatty acid oxidation, and cholesterol metabolism. Glycerol-3-phosphate acyltransferases (GPAT) catalyze the initial step in glycerolipid synthesis, and sterol regulatory element binding protein (SREBP-1c), which is induced by insulin, regulates genes required for glucose metabolism and fatty acid and lipid production. Hepatic lipogenesis is similar to adipocyte lipogenesis and involves therefore similar key enzymes such as AMP-activated protein kinase (AMPK), SREBP-1, acetyl-CoA carboxylation (ACC), HMG-CoA reductase (HMGCR), fatty acid synthase (FAS) and PPARα [173]. ACC catalyzes the carboxylation of acetyl-CoA to malonyl-CoA, but also controls fatty acid oxidation by means of the ability of malonyl-CoA to inhibit carnitine palmitoyltransferase I (CPT-1), the rate-limiting step in fatty acid uptake and oxidation by mitochondria. PPAR-α is a nuclear receptor that promotes uptake, utilization, and catabolism of fatty acids by upregulation of genes involved in fatty acid transport, fatty acid binding and activation, and peroxisomal and mitochondrial fatty acid β-oxidation.

Besides their role in lipogenesis, ACC and HMGCR are involved in cholesterol production in the hepatocytes. This cholesterol, together with triglycerides, are embedded in lipoproteins, mainly APO-100 [174], APO-C and APO-E, to form VLDL that, after lipoprotein lipase action, delivers the triglycerides to the adipose tissue and becomes LDL. This LDL is considered as “bad cholesterol”, as the excess of LDL is absorbed by macrophages which may form an atherosclerotic plaque in the blood vessel. Cholesterol is recycled in the body after binding of LDL and HDL, consisting of mainly APO-A1, to lipoprotein receptors in the hepatocytes.

The HepG2 cell line is by far the most studied hepatic cell line [175]. It is a continuous cell line derived from a hepatocellular carcinoma, grows as a monolayer of epithelium and in aggregates, are polarized cells and secrete many plasma proteins including transferrin, fibrinogen, plasminogen and albumin. It has a high predictive value for biotransformation processes by the liver [175]. HepG2 cells have been cultivated in single cell culture systems, as well as in co-culture with other cell lines such as the Caco-2 cell line, and in 3D conformation with enhanced cell structure and functional properties [176].

Based on Table 1, it can be seen that especially lipid and cholesterol metabolism in hepatocytes is strongly influenced by a wide variety of polyphenols. Particularly, flavonoids including quercetin, resveratrol, epicatechin and cyanidin, and phenolic acids including ellagic and gallic acid are shown to regulate lipid and cholesterol metabolism. Eventually, polyphenols have potential to heal liver disorders related to cardiovascular diseases.

#### 3.2.5. The Immune System

The immune system is a system of biological structures and processes within an organism that protects against diseases and can be modified by diet, pharmacologic agents, environmental pollutants, and naturally occurring food substances, such as polyphenols [177]. Immunomodulatory signals such as IL-1β, IL-6, IL-8, IL-10, NF-κB, and TNFα, can be produced by most of the tissues in the body—as discussed before—and they are also expressed in specific white blood cells, such as macrophages, mast cells, neutrophils, T cells and B cells [178]. Atherosclerosis, the major cause of cardiovascular disease (CVD), is a chronic inflammatory condition whereby immune competent white blood cells are infiltrated in atherosclerotic plaques where they produce mainly pro-inflammatory cytokines [179]. In those lesions, oxidized LDL and dead cells cause inflammation and immune stimulation. The antioxidant capacity of polyphenols can therefore modulate the immune system in various ways, mainly through the inhibition of enzymes related to inflammation such as cyclooxygenase (COX) [136,137,138,139] and key regulatory transcription factors including peroxisome proliferator-activated receptors (PPAR) [136] and nitric oxide synthase (NOS) [81].

The THP-1 cell line is the most commonly used cell line to study immune response. It is a human-derived monocytic cell line derived from an acute monocytic leukemia patient. After treatment with phorbol esters, THP-1 cells differentiate into macrophage-like cells. Compared to other human myeloid cell lines, such as U937 cells, differentiated THP-1 cells behave more like native monocyte-derived macrophages. Therefore, the THP-1 cell line is a suitable model for studying the mechanisms involved in macrophage differentiation, and for exploring the regulation of macrophage-specific genes [180]. The RAW 264.7 is a murine cell line with similar properties, and the NR8383 cell line is an alveolar cell line with macrophage-like properties from rat origin. The HMC-1 cell line is a mast cell line of human origin.

Several polyphenols have been shown to stimulate immune responses in these cell lines, as well as factors affecting vasorelaxation, proliferation and apoptosis, cell migration and energy metabolism. It has been shown that resveratrol, grape seed procyanidins, quince peel polyphenols, naringenin chalcone, epicatechin, caffeic acid, curcumin and cyanidin-3-*O*-β-glucoside inhibit the inflammatory cytokines (Table 1).

#### 3.2.6. Overall Effect

From Table 1, it can be concluded that polyphenols modulate intestine, adipose tissue, endothelium, liver and immune system through various different metabolisms and consequently contribute to the prevention of cardiovascular diseases. Most of these studies have been carried out on resveratrol, catechins, and anthocyanins. Resveratrol affects these target tissues mostly through modification of energy metabolism and inhibition of inflammatory markers. Similarly, the impact of catechins on these tissues was also largely based on the regulation of energy metabolism. In addition, catechins are shown to have significant effects on cell proliferation. Furthermore, several anthocyanins may regulate the energy metabolism. In particular, they are shown to have significant effects on the liver. Overall, although the exact behavior by which polyphenols produce their effects is not fully understood, it has been demonstrated that they have the potential to improve diseases related to cardiovascular health.

One major concern for the use of cell cultures for the study of biomarkers triggered by polyphenols is the cancer-related origin of many commercially available cell cultures as many polyphenols selectively induce apoptosis in cancer cells by deregulation of the cell cycle, and are therefore considered as potential anticancer agents [181]. Polyphenols can act as either antioxidant or prooxidant, depending on the dose, cell type and cell culture conditions. In general, most bioactive actions are related to the reactive oxygen species (ROS) scavenging potential of the polyphenols, including cardiovascular effects such as hypertension [182]. In contrast, their anticancer effect has been shown to be mediated through their prooxidant properties, as cancer cells have higher and more persistent oxidative stress levels compared to normal cells, which makes them more sensitive towards the extra ROS levels generated by pro-oxidants. In a recent study by Sak [181], the cytotoxicity, expressed as IC50 values, of flavonoids on more than 150 cell lines from bladder, blood, bone, breast, colon, liver, lung, melanoma, mouth, esophagus, ovary, pancreas, prostate, stomach and uterus origin, was reviewed. It was concluded that the toxicity effect was highly variable and dependent on flavonoid type, dose, cell line origin, and expression of estrogen receptors.

Besides the origin of the cells, one may also question whether the observed changes in biomarkers/cytokines in response to the polyphenol are considered to be beneficial or adverse. This is hard to assess, because this is strongly dependent on (i) the concentration of the added compound, (ii) the duration of incubation; (iii) the intensity of the cellular response in terms of amount of “marker” that is produced and (iii) the pathways that are affected by the “marker”, which is on itself also dependent on the amount of “marker” that is produced. So far, the relevant dose that needs to be added to a cell is still under discussion, and also the mode-of-action of the polyphenols, of which “anti-oxidant” activity is an example, is not fully understood. Besides that, only few proteomics, transcriptomics and metabolomics studies have been performed to have a full picture of the mechanisms. Therefore, although cell models provide a useful tool to perform mechanistic research, they should always be compared with *in vivo* data or primary cells.

## 4. Current Cell Culture Research: Trends and Potential Application for Polyphenol Research

The past five years, cell culture research has evolved towards the development of more complex models, to obtain more relevant models that allow investigation of inter-cell signaling and cytokine expression. The Transwell^®^ system, a static double well system separated by a filter membrane and generally used for transport experiments, and the collagen-embedded cell setup, are widely applied for indirect contact co-cultivation of multiple cell types. Nowadays, research groups and companies focus on the cultivation of one or more cell types on carriers and scaffolds containing extracellular matrix compounds to allow spatial organization and enhanced differentiation of the cells [183]. In some specific setups, low shear stress conditions are applied on cells adhered to carriers in rotating wall vessels, which may result in the differentiation of one cell line to multiple phenotypes (for instance HT-29 colonic cell line to the enterocyte and mucin producing phenotype). In other setups, dynamic conditions are applied to allow longer viability of the cells. In general, cell morphology and metabolism in these more advanced setups are now characterized and validated with tissue samples, and a first attempt to investigate the effects of highly characterized drugs was made. Yet, they are not widely applied for screening purposes of (digested) nutrients because of the specific expertise that is required.

As illustrated by Table 2, co-culture models of intestinal cell lines (mainly the Caco-2 cell line) with other intestinal cell lines, liver, endothelial, adipocyte, neuronal, fibroblast and a variety of immune cells have been developed. Co-cultures of the Caco-2 and the intestinal HT29-MTX cell line has been used to investigate the impact mucins on the bioavailability of curcumin nanoparticles [184]. Especially co-cultures of intestinal cell lines with immune cells became very popular for the investigation of the effect of pathogens, probiotics, lipopolysaccharides, and a limited amount of environmental and food contaminants on intestinal behavior and general health (Table 2). Less literature is available about co-cultures of intestinal cells with endothelial cells and only recently, the first publication about co-culture of intestinal with brown adipose tissue cell lines has appeared [185]. A co-culture model of differentiated Caco-2 cells with primary HUVEC and HMEC-1 cells has been published in the context of ICAM-1 and VCAM-1 expression through a NF-κB-mediated mechanism [186]. In a publication of Zgouras *et al.*, (2003) [187], undifferentiated Caco-2 cells were combined with HUVEC cells to investigate the effect of butyrate on tumor-derived angiogenesis. Only few of these systems have been used for the investigation of the impact of polyphenols on the cross-talk between intestinal and endothelial cell types in the context of cardiovascular diseases. In a publication of Kuntz *et al.*, (2015) [188], a mixture of Caco-2 cells with mucus secreting HT29-B6 cells was co-cultured with HUVEC cells, and it was shown that addition of an anthocyanin-rich grape extract had a beneficial effect on inflammation inhibition in the context of atherosclerosis, as measured by ICAM-1, VCAM-1, IL-6, IL-8 and E-selectin levels. Similarly, in another study the beneficial effect of resveratrol on NO production, oxidative stress and production of VEGF, ICAM-1 and IL-8 in a co-culture system of Caco-2 cells with the EA.hy92 6 cell line was demonstrated (unpublished data).

**Table 2:** Co-culture models.

	Intestinal Cell Lines	Co-Cultured Cell (Line)	Experimental Setup	Application	Ref.
Intestine	Caco-2, Caco-2BBE	HT-29, HT-29-MTX, M-cells	Direct contact	Iron bioavailbaility, breast milk effects, nanoparticle uptake, curcumin bioavailability	[184,189,190,191,192,193]
Liver	Caco-2; Caco-2-TC7	HepG2, HepaRG, murine 3A	Transwell and continuous perfused fluidic system	Benzo-a-pyrene toxicity, b-carotene and retinoid transport	[194,195,196]
Neuronal	Caco-2, HT-29	PC12, glial cells, primary enteric neurocytes	Collagen-embedded system, Transwell system	Co-culture characteristics, LPS stimulation, pathogen invasion	[197,198,199,200]
Fibroblast	Caco-2, IEC-6, IPI-21, CRL-2102	Primary human and rat fibroblasts, Rat-2	Collagen-embedded, long term 3D	Co-culture characteristics	[201,202,203,204]
Immune cells	Caco-2; HT-29, m-ICcl2	Whole blood cells, dendritic cells from isolated blood monocytes and bone marrow, lymphoblastoic TK6 cells, macrophage-like THP-1 and RAW264.7, murine lymphocytes of Peyers patches, Jurkat cells, RBL-2H3 (rat basophils), mast cells	Transwell system, floating filter system and direct contact, indirect micropattern surface	Co-culture characteristics, bioactivity of drugs, LPS, probiotica, benzo-a-pyrene, aflatoxin, fucoidan, immunoreactivity of ovalbumin	[205,206,207,208,209,210,211,212,213,214,215,216]
3 or more cell types	Caco-2+HT29-MTX	Raji B, fibroblast + immunocytes, blood derived macrophages + dendritic cells	Transwell system, direct contact, collagen-embedded Transwell system,	(Peptide) drug transport and permeability	[217,218,219]
Adipocyte	Caco-2, HT29-19A	PAZ-6	Transwell system	Co-culture characteristics	[185]
Endothelium	Caco-2, HT29-6B, LS180EB3	Primary HMEC, immortalized isolated HMEC from lymph node, appendix, lung, skin and intestine microvessels, HUVEC, EA.hy926 cells	Transwell system, 3D dynamic model with decellularized jejunum segments, indirect contact	Co-culture characteristics, migration and adhesion of tumor cells, effect of anthocyanins of grape	[220,221]
	**Adipocyte Cells**	**Co-cultured Cell (Line)**	**Experimental Setup**	**Application**	**Ref.**
Immune cells	Mouse preadipocytes, 3T3-L1	RAW264	Direct contact	Cross-talk grape, Maqui, calafate, blueberry polyphenol extracts, naringenin chalcone	[81,222,223]

LPS: lipopolysaccharide.

The impact of polyphenols on other co-culture models, which are not including intestinal transport or modifications, have also been published before. In co-culture models of adipocyte cell lines with immune cells such as macrophages [81,222,223], as well as with endothelial cells have been more studied in the context of polyphenol research. Different crosstalk mechanisms including ROS, inflammatory markers, MCP-1 and PAI-1 were influenced by wine, maqui, calafate and blueberry polyphenol extract as well as narigenin chalcone, in a mouse adipocyte-macrophage co-culture system. Although the primary objective of these models was related to the effect of polyphenols on obesity, these cross-talk mechanisms may have an indirect impact on endothelial function as well.

Finally, very recent research demonstrates the link of cardiovascular diseases with the intestinal microbial composition and metabolism [224,225], although this has still been far less studied than the microbial impact on metabolic syndrome, obesity and diabetes [226]. As polyphenols have been shown to have a strong influence on microbial metabolism, as well as on microbial composition due to their antimicrobial effects, we consider this field to be largely unexplored. Single cell models and Transwell models have been widely applied in combination with intestinal microbiota to investigate adhesion of probiotic bacteria and pathogens [227], transient intestinal colonization [228], modulation of the immune system [229], and healing of damaged intestinal mucosa [230]. The major drawback of using conventional (Transwell) systems with live bacteria is the limited contact time (only a few hours) and the sensitivity of the cells towards intestinal fluids. New dynamic modules, such as rotating wall vessel production units, the Host-Microbe Interaction model [231] and the gut-on-a-chip model [232], allow increased differentiation of the intestinal cell lines, longer co-incubation times and biofilm formation. Therefore, several of these models could be useful for the study of the impact of polyphenols on the crosstalk mechanisms not only between host cells individually, but also between the host and the residing microbial community, in the context of cardiovascular diseases.

## 5. General Conclusions

This review has demonstrated that cell lines, almost exclusively in a monoculture setup, have widely been used to understand the mechanisms by which polyphenols may exert effects on cardiovascular parameters. Yet, though cell lines offer an easy-to-use and high-throughput model to screen and rank biophenols according to their bioavailability and bioactivity, one may question the relevance of these models to be extrapolated to the *in vivo* situation. Firstly, the concentration of polyphenols applied in these studies is often a factor 10 to 1000 higher than what is circulating in the blood stream. Next to this, the cancer origin of many commercially available cell lines, as well as the micro-environment in which the cells are isolated and cultivated outside the host may strongly impact their gene expression profile, and hence also their response to (lower concentrations) of polyphenols. *In vivo*, this micro-environment can be considered as a cocktail of many chemicals structures that find their origin in the ingested foods, metabolites from enzymatic, microbial and cellular origin, cytokines, extracellular matrix compounds and circulating blood factors. In addition, it was already proven that mechanical factors such as low shear stress—present in the gut lumen as well as in the blood stream—strongly impact the differentiation behavior of the cells. So far, the majority of these cell studies do not take into account this micro-environment, or only use a limited set of stressors to simulate the onset of cardiovascular disease. The lack of these studies are probably the result of (i) the lack of analytical data about the exact composition of this micro-environment; (ii) the lack of protocols for stabilization of the micro-environment to limit the variability in cell response; and (iii) the lack of protocols describing how to apply the micro-environment to the cells in a successful way. Recent progress in the -omics areas together with the design of more complex cell models may trigger more relevant mechanistic research in this field.

## References

[1] D’Archivio M., Filesi C., Vari R., Scazzocchio B., Masella R. (2010). Bioavailability of the polyphenols: Status and controversies. Int. J. Mol. Sci..

[2] Ignat I., Volf I., Popa V.I. (2011). A critical review of methods for characterisation of polyphenolic compounds in fruits and vegetables. Food Chem..

[3] Balasundram N., Sundram K., Samman S. (2006). Phenolic compounds in plants and agri-industrial by-products: Antioxidant activity, occurrence, and potential uses. Food Chem..

[4] Spencer J.P., Abd El Mohsen M.M., Minihane A.M., Mathers J.C. (2008). Biomarkers of the intake of dietary polyphenols: Strengths, limitations and application in nutrition research. Br. J. Nutr..

[5] Pandey K., Rizvi S. (2009). Plant polyphenols as dietary antioxidants in human health and disease. Oxid. Med. Cell Longev..

[6] Manach C., Scalbert A., Morand C., Remesy C., Jimenez L. (2004). Polyphenols: Food sources and bioavailability. Am. J. Clin. Nutr..

[7] D’Archivio M., Filesi C., Di Benedetto R., Gargiulo R., Giovannini C., Masella R. (2007). Polyphenols, dietary sources and bioavailability. Ann.Ist. Super. Sanita.

[8] Lenucci M.S., Cadinu D., Taurino M., Piro G., Dalessandro G. (2006). Antioxidant composition in cherry and high-pigment tomato cultivars. J. Agric. Food Chem..

[9] Carpene C., Gomez-Zorita S., Deleruyelle S., Carpene M.A. (2015). Novel strategies for preventing diabetes and obesity complications with natural polyphenols. Curr. Med. Chem..

[10] Ryan L., Prescott S.L. (2010). Stability of the antioxidant capacity of twenty-five commercially available fruit juices subjected to an *in vitro* digestion. Int. J. Food Sci. Technol..

[11] Parada J., Aguilera J.M. (2007). Food microstructure affects the bioavailability of several nutrients. J. Food Sci..

[12] Porrini M., Riso P. (2008). Factors influencing the bioavailability of antioxidants in foods: A critical appraisal. Nutr. Metab. Cardiovasc. Dis..

[13] Manach C., Williamson G., Morand C., Scalbert A., Remesy C. (2005). Bioavailability and bioefficacy of polyphenols in humans. I. Review of 97 bioavailability studies. Am. J. Clin. Nutr..

[14] Kishimoto Y., Tani M., Kondo K. (2013). Pleiotropic preventive effects of dietary polyphenols in cardiovascular diseases. Eur. J. Clin. Nutr..

[15] Tsang C., Higgins S., Duthie G.G., Duthie S.J., Howie M., Mullen W., Lean M.E.J., Crozier A. (2005). The influence of moderate red wine consumption on antioxidant status and indices of oxidative stress associated with chd in healthy volunteers. Br. J. Nutr..

[16] Giongo L., Bozza E., Caciagli P., Valente E., Pasquazzo M.T., Pedrolli C., Iorio E.L., Costa A. (2011). Short-term blueberry intake enhances biological antioxidant potential and modulates inflammation markers in overweight and obese children. J. Berry Res..

[17] Estruch R., Sacanella E., Badia E., Antunez E., Nicolas J.M., Fernandez-Sola J., Rotilio D., de Gaetano G., Rubin E., Urbano-Marquez A. (2004). Different effects of red wine and gin consumption on inflammatory biomarkers of atherosclerosis: Aprospective randomized crossover trial. Effects of wine on inflammatory markers. Atherosclerosis.

[18] Micallef M., Lexis L., Lewandowski P. (2007). Red wine consumption increases antioxidant status and decreases oxidative stress in the circulation of both young and old humans. Nutr. J..

[19] Alvarez-Suarez J.M., Giampieri F., Tulipani S., Casoli T., di Stefano G., González-Paramás A.M., Santos-Buelga C., Busco F., Quiles J.L., Cordero M.D. (2014). One-month strawberry-rich anthocyanin supplementation ameliorates cardiovascular risk, oxidative stress markers and platelet activation in humans. J. Nutr. Biochem..

[20] Napoli R., Cozzolino D., Guardasole V., Angelini V., Zarra E., Matarazzo M., Cittadini A., Sacca L., Torella R. (2005). Red wine consumption improves insulin resistance but not endothelial function in type 2 diabetic patients. Metabolism.

[21] Tousoulis D., Ntarladimas I., Antoniades C., Vasiliadou C., Tentolouris C., Papageorgiou N., Latsios G., Stefanadis C. (2008). Acute effects of different alcoholic beverages on vascular endothelium, inflammatory markers and thrombosis fibrinolysis system. Clin. Nutr..

[22] Vinson J.A., Teufel K., Wu N. (2001). Red wine, dealcoholized red wine, and especially grape juice, inhibit atherosclerosis in a hamster model. Atherosclerosis.

[23] Waddington E., Puddey I.B., Croft K.D. (2004). Red wine polyphenolic compounds inhibit atherosclerosis in apolipoprotein E-deficient mice independently of effects on lipid peroxidation. Am. J. Clin. Nutr..

[24] Mineharu Y., Koizumi A., Wada Y., Iso H., Watanabe Y., Date C., Yamamoto A., Kikuchi S., Inaba Y., Toyoshima H. (2011). Coffee, green tea, black tea and oolong tea consumption and risk of mortality from cardiovascular disease in japanese men and women. J. Epidemiol. Commun. Health.

[25] de Koning Gans J.M., Uiterwaal C.S., van der Schouw Y.T., Boer J.M., Grobbee D.E., Verschuren W.M., Beulens J.W. (2010). Tea and coffee consumption and cardiovascular morbidity and mortality. Arterioscler. Thromb. Vasc. Biol..

[26] Vinson J.A., Teufel K., Wu N. (2004). Green and black teas inhibit atherosclerosis by lipid, antioxidant, and fibrinolytic mechanisms. J. Agric. Food Chem..

[27] Potter S.M., Baum J.A., Teng H.Y., Stillman R.J., Shay N.F., Erdman J.W. (1998). Soy protein and isoflavones: Their effects on blood lipids and bone density in postmenopausal women. Am. J. Clin. Nutr..

[28] Adams M.R., Golden D.L., Register T.C., Anthony M.S., Hodgin J.B., Maeda N., Williams J.K. (2002). The atheroprotective effect of dietary soy isoflavones in apolipoprotein E−/− mice requires the presence of estrogen receptor-α. Arterioscler. Thromb. Vasc. Biol..

[29] Bowey E., Adlercreutz H., Rowland I. (2003). Metabolism of isoflavones and lignans by the gut microflora: A study in germ-free and human flora associated rats. Food Chem. Toxicol..

[30] Cardona F., Andres-Lacueva C., Tulipani S., Tinahones F.J., Queipo-Ortuno M.I. (2013). Benefits of polyphenols on gut microbiota and implications in human health. J. Nutr. Biochem..

[31] Chiva-Blanch G., Visioli F. (2012). Polyphenols and health: Moving beyond antioxidants. J. Berry Res..

[32] Forbes-Hernandez T.Y., Gasparrini M., Afrin S., Bompadre S., Mezzetti B., Quiles J.L., Giampieri F., Battino M. (2015). The healthy effects of strawberry polyphenols: Which strategy behind antioxidant capacity?. Crit. Rev. Food Sci. Nutr..

[33] Friedrich M., Petzke K.J., Raederstorff D., Wolfram S., Klaus S. (2012). Acute effects of epigallocatechin gallate from green tea on oxidation and tissue incorporation of dietary lipids in mice fed a high-fat diet. Int. J. Obes..

[34] Qin B.L., Dawson H.D., Schoene N.W., Polansky M.M., Anderson R.A. (2012). Cinnamon polyphenols regulate multiple metabolic pathways involved in insulin signaling and intestinal lipoprotein metabolism of small intestinal enterocytes. Nutrition.

[35] Denis M.C., Furtos A., Dudonne S., Montoudis A., Garofalo C., Desjardins Y., Delvin E., Levy E. (2013). Apple peel polyphenols and their beneficial actions on oxidative stress and inflammation. PLoS ONE.

[36] Rosillo M.A., Sanchez-Hidalgo M., Cárdeno A., Alarcón de la Lastra C. (2011). Protective effect of ellagic acid, a natural polyphenolic compound, in a murine model of Crohn’s disease. Biochem. Pharmacol..

[37] Angel-Morales G., Noratto G., Mertens-Talcott S. (2012). Red wine polyphenolics reduce the expression of inflammation markers in human colon-derived CCD-18CO myofibroblast cells: Potential role of microRNA-126. Food Funct..

[38] Gessner D.K., Ringseis R., Siebers M., Keller J., Kloster J., Wen G., Eder K. (2012). Inhibition of the pro-inflammatory NF-κB pathway by a grape seed and grape marc meal extract in intestinal epithelial cells. J. Anim. Physiol. Anim. Nutr..

[39] Song Y.A., Park Y.L., Yoon S.H., Kim K.Y., Cho S.B., Lee W.S., Chung I.J., Joo Y.E. (2011). Black tea polyphenol theaflavin suppresses LPS-induced ICAM-1 and VCAM-1 expression via blockage of NF-kB and JNK activation in intestinal epithelial cells. Inflamm. Res..

[40] Romier-Crouzet B., Van De Walle J., During A., Joly A., Rousseau C., Henry O., Larondelle Y., Schneider Y.-J. (2009). Inhibition of inflammatory mediators by polyphenolic plant extracts in human intestinal Caco-2 cells. Food Chem. Toxicol..

[41] Ruiz P.A., Haller D. (2006). Functional diversity of flavonoids in the inhibition of the proinflammatory NF-κB, IRF, and Akt signaling pathways in murine intestinal epithelial cells. J. Nutr..

[42] Yang F., Oz H.S., Barve S., de Villiers W.J.S., McClain C.J., Varilek G.W. (2001). The green tea polyphenol (−)-epigallocatechin-3-gallate blocks nuclear factor-κB activation by inhibiting IκB kinase activity in the intestinal epithelial cell line IEC-6. Mol. Pharmacol..

[43] Leifert W.R., Abeywardena M.Y. (2008). Grape seed and red wine polyphenol extracts inhibit cellular cholesterol uptake, cell proliferation, and 5-lipoxygenase activity. Nutr. Res..

[44] Kim B., Park Y., Wegner C.J., Bolling B.W., Lee J. (2013). Polyphenol-rich black chokeberry (*Aronia melanocarpa*) extract regulates the expression of genes critical for intestinal cholesterol flux in Caco-2 cells. J. Nutr. Biochem..

[45] Haas M.J., Onstead-Haas L.M., Szafran-Swietlik A., Kojanian H., Davis T., Armstrong P., Wong N.C.W., Mooradian A.D. (2014). Induction of hepatic apolipoprotein A-I gene expression by the isoflavones quercetin and isoquercetrin. Life Sci..

[46] Drira R., Chen S., Sakamoto K. (2011). Oleuropein and hydroxytyrosol inhibit adipocyte differentiation in 3T3-L1 cells. Life Sci..

[47] Warnke I., Goralczyk R., Fuhrer E., Schwager J. (2011). Dietary constituents reduce lipid accumulation in murine C3H10 T1/2 adipocytes: A novel fluorescent method to quantify fat droplets. Nutr. Metab..

[48] Lee Y., Bae E.J. (2013). Inhibition of mitotic clonal expansion mediates fisetin-exerted prevention of adipocyte differentiation in 3T3-L1 cells. Arch. Pharmacal Res..

[49] Gomez-Zorita S., Treguer K., Mercader J., Carpene C. (2013). Resveratrol directly affects *in vitro* lipolysis and glucose transport in human fat cells. J. Physiol. Biochem..

[50] Park H.J., Chung B.Y., Lee M.K., Song Y., Lee S.S., Chu G.M., Kang S.N., Song Y.M., Kim G.S., Cho J.H. (2012). Centipede grass exerts anti-adipogenic activity through inhibition of C/EBPβ, C/EBPα, and PPARγ expression and the Akt signaling pathway in 3T3-L1 adipocytes. BMC Complement. Altern. Med..

[51] Rosenow A., Noben J.-P., Jocken J., Kallendrusch S., Fischer-Posovszky P., Mariman E.C.M., Renes J. (2012). Resveratrol-induced changes of the human adipocyte secretion profile. J. Proteome Res..

[52] Freise C., Trowitzsch-Kienast W., Erben U., Seehofer D., Kim K.Y., Zeitz M., Ruehl M., Somasundaram R. (2013). (+)-episesamin inhibits adipogenesis and exerts anti-inflammatory effects in 3T3-L1 (pre)adipocytes by sustained WNT signaling, down-regulation of PPARγ and induction of iNOS. J. Nutr. Biochem..

[53] Min S.Y., Yang H., Seo S.G., Shin S.H., Chung M.Y., Kim J., Lee S.J., Lee H.J., Lee K.W. (2013). Cocoa polyphenols suppress adipogenesis *in vitro* and obesity *in vivo* by targeting insulin receptor. Int. J. Obes..

[54] Gosmann G., Barlette A.G., Dhamer T., Arcari D.P., Santos J.C., de Camargo E.R., Acedo S., Gambero A., Gnoatto S.C., Ribeiro M.L. (2012). Phenolic compounds from mate (*ilex paraguariensis*) inhibit adipogenesis in 3T3-L1 preadipocytes. Plant Foods Hum. Nutr..

[55] Moghe S.S., Juma S., Imrhan V., Vijayagopal P. (2012). Effect of blueberry polyphenols on 3T3-F442a preadipocyte differentiation. J. Med. Food.

[56] Lin J., Della-Fera M.A., Baile C.A. (2005). Green tea polyphenol epigallocatechin gallate inhibits adipogenesis and induces apoptosis in 3T3-L1 adipocytes. Obes. Res..

[57] Okla M., Kang I., Kim D.M., Gourineni V., Shay N., Gu L., Chung S. (2015). Ellagic acid modulates lipid accumulation in primary human adipocytes and human hepatoma Huh7 cells via discrete mechanisms. J. Nutr. Biochem..

[58] Kim S., Jin Y., Choi Y., Park T. (2011). Resveratrol exerts anti-obesity effects via mechanisms involving down-regulation of adipogenic and inflammatory processes in mice. Biochem. Pharmacol..

[59] Beaudoin M.S., Snook L.A., Arkell A.M., Simpson J.A., Holloway G.P., Wright D.C. (2013). Resveratrol supplementation improves white adipose tissue function in a depot-specific manner in Zucker diabetic fatty rats. Am. J. Physiol. Regul. Integr. Comp. Physiol..

[60] Claussnitzer M., Skurk T., Hauner H., Daniel H., Rist M.J. (2011). Effect of flavonoids on basal and insulin-stimulated 2-deoxyglucose uptake in adipocytes. Mol. Nutr. Food Res..

[61] Saito T., Abe D., Sekiya K. (2008). Sakuranetin induces adipogenesis of 3T3-L1 cells through enhanced expression of PPARγ2. Biochem. Biophys. Res. Commun..

[62] Lee H.-H., Kim K.-J., Lee O.-H., Lee B.-Y. (2010). Effect of pycnogenol® on glucose transport in mature 3T3-L1 adipocytes. Phytother. Res..

[63] Scazzocchio B., Varì R., Filesi C., D’Archivio M., Santangelo C., Giovannini C., Iacovelli A., Silecchia G., Volti G.L., Galvano F. (2011). Cyanidin-3-*O*-β-glucoside and protocatechuic acid exert insulin-like effects by upregulating PPARγ activity in human omental adipocytes. Diabetes.

[64] Zingg J.M., Hasan S.T., Meydani M. (2013). Molecular mechanisms of hypolipidemic effects of curcumin. Biofactors.

[65] Boque N., de la Iglesia R., de la Garza A.L., Milagro F.I., Olivares M., Banuelos O., Soria A.C., Rodriguez-Sanchez S., Martinez J.A., Campion J. (2013). Prevention of diet-induced obesity by apple polyphenols in wistar rats through regulation of adipocyte gene expression and DNA methylation patterns. Mol. Nutr. Food Res..

[66] Tian C., Ye X., Zhang R., Long J., Ren W., Ding S., Liao D., Jin X., Wu H., Xu S. (2013). Green tea polyphenols reduced fat deposits in high fat-fed rats via Erk1/2-PPARγ-adiponectin pathway. PLoS ONE.

[67] Chuang C.C., Bumrungpert A., Kennedy A., Overman A., West T., Dawson B., McIntosh M.K. (2011). Grape powder extract attenuates tumor necrosis factor α-mediated inflammation and insulin resistance in primary cultures of human adipocytes. J. Nutr. Biochem..

[68] Santiago-Mora R., Casado-Diaz A., De Castro M.D., Quesada-Gomez J.M. (2011). Oleuropein enhances osteoblastogenesis and inhibits adipogenesis: The effect on differentiation in stem cells derived from bone marrow. Osteoporos. Int..

[69] Shin D.W., Kim S.N., Lee S.M., Lee W., Song M.J., Park S.M., Lee T.R., Baik J.-H., Kim H.K., Hong J.-H. (2009). (−)-Catechin promotes adipocyte differentiation in human bone marrow mesenchymal stem cells through PPARγ transactivation. Biochem. Pharmacol..

[70] Harmon A.W., Harp J.B. (2001). Differential effects of flavonoids on 3T3-L1 adipogenesis and lipolysis. Am. J. Physiol. Cell Physiol..

[71] Hu P., Zhao L., Chen J.G. (2015). Physiologically achievable doses of resveratrol enhance 3T3-L1 adipocyte differentiation. Eur. J. Nutr..

[72] Ahn J., Lee H., Kim S., Ha T. (2010). Curcumin-induced suppression of adipogenic differentiation is accompanied by activation of WNT/β-catenin signaling. Am. J. Physiol. Cell Physiol..

[73] Ogasawara J., Kitadate K., Nishioka H., Fujii H., Sakurai T., Kizaki T., Izawa T., Ishida H., Ohno H. (2011). Comparison of the effect of oligonol, a new lychee fruit-derived low molecular form of polyphenol, and epigallocatechin-3-gallate on lipolysis in rat primary adipocytes. Phytother. Res..

[74] Ogasawara J., Kitadate K., Nishioka H., Fujii H., Sakurai T., Kizaki T., Izawa T., Ishida H., Tanno M., Ohno H. (2010). Oligonol, an oligomerized lychee fruit-derived polyphenol, activates the Ras/Raf-1/MEK1/2 cascade independent of the IL-6 signaling pathway in rat primary adipocytes. Biochem. Biophys. Res. Commun..

[75] Chen S.F., Zhou N.M., Zhang Z.L., Li W.X., Zhu W. (2015). Resveratrol induces cell apoptosis in adipocytes via AMPK activation. Biochem. Biophys. Res. Commun..

[76] Yoshimura Y., Nishii S., Zaima N., Moriyama T., Kawamura Y. (2013). Ellagic acid improves hepatic steatosis and serum lipid composition through reduction of serum resistin levels and transcriptional activation of hepatic PPARα in obese, diabetic KK-Ay mice. Biochem. Biophys. Res. Commun..

[77] Mercader J., Palou A., Bonet M.L. (2011). Resveratrol enhances fatty acid oxidation capacity and reduces resistin and retinol-binding protein 4 expression in white adipocytes. J. Nutr. Biochem..

[78] Tome-Carneiro J., Gonzalvez M., Larrosa M., Yanez-Gascon M.J., Garcia-Almagro F.J., Ruiz-Ros J.A., Tomas-Barberan F.A., Garcia-Conesa M.T., Espin J.C. (2013). Grape resveratrol increases serum adiponectin and downregulates inflammatory genes in peripheral blood mononuclear cells: A triple-blind, placebo-controlled, one-year clinical trial in patients with stable coronary artery disease. Cardiovasc. Drugs Ther..

[79] Wang A., Liu M., Liu X., Dong L.Q., Glickman R.D., Slaga T.J., Zhou Z., Liu F. (2011). Up-regulation of adiponectin by resveratrol: The essential roles of the Akt/FOXO1 and AMP-activated protein kinase signaling pathways and Dsba-L. J. Biol. Chem..

[80] Sharma S., Misra C.S., Arumugam S., Roy S., Shah V., Davis J.A., Shirumalla R.K., Ray A. (2011). Antidiabetic activity of resveratrol, a known SIRT1 activator in a genetic model for type-2 diabetes. Phytother. Res..

[81] Hirai S., Kim Y., Goto T., Kang M.-S., Yoshimura M., Obata A., Yu R., Kawada T. (2007). Inhibitory effect of naringenin chalcone on inflammatory changes in the interaction between adipocytes and macrophages. Life Sci..

[82] Cullberg K.B., Olholm J., Paulsen S.K., Foldager C.B., Lind M., Richelsen B., Pedersen S.B. (2013). Resveratrol has inhibitory effects on the hypoxia-induced inflammation and angiogenesis in human adipose tissue *in vitro*. Eur. J. Pharm. Sci..

[83] Cao H., Anderson R.A. (2011). Cinnamon polyphenol extract regulates tristetraprolin and related gene expression in mouse adipocytes. J. Agric. Food Chem..

[84] Mojzis J., Varinska L., Mojzisova G., Kostova I., Mirossay L. (2008). Antiangiogenic effects of flavonoids and chalcones. Pharmacol. Res..

[85] Duluc L., Soleti R., Clere N., Andriantsitohaina R., Simard G. (2012). Mitochondria as potential targets of flavonoids: Focus on adipocytes and endothelial cells. Curr. Med. Chem..

[86] Klinge C.M., Wickramasinghe N.S., Ivanova M.M., Dougherty S.M. (2008). Resveratrol stimulates nitric oxide production by increasing estrogen receptor α-Src-caveolin-1 interaction and phosphorylation in human umbilical vein endothelial cells. FASEB J..

[87] Silambarasan T., Manivannan J., Priya M.K., Suganya N., Chatterjee S., Raja B. (2014). Sinapic acid prevents hypertension and cardiovascular remodeling in pharmacological model of nitric oxide inhibited rats. PLoS ONE.

[88] Dong J., Xu X., Liang Y., Head R., Bennett L. (2011). Inhibition of angiotensin converting enzyme (ACE) activity by polyphenols from tea (*Camellia sinensis*) and links to processing method. Food Funct..

[89] Rivas-Arreola M.J., Rocha-Guzman N.E., Gallegos-Infante J.A., Gonzalez-Laredo R.F., Rosales-Castro M., Bacon J.R., Cao R., Proulx A., Intriago-Ortega P. (2010). Antioxidant activity of oak (*Quercus*) leaves infusions against free radicals and their cardioprotective potential. Pakistan J. Biol. Sci..

[90] Persson I.A., Persson K., Andersson R.G. (2009). Effect of vaccinium myrtillus and its polyphenols on angiotensin-converting enzyme activity in human endothelial cells. J. Agric. Food Chem..

[91] Olszanecki R., Bujak-Gizycka B., Madej J., Suski M., Wolkow P.P., Jawien J., Korbut R. (2008). Kaempferol, but not resveratrol inhibits angiotensin converting enzyme. J. Physiol. Pharmacol..

[92] Kang D.G., Kim Y.C., Sohn E.J., Lee Y.M., Lee A.S., Yin M.H., Lee H.S. (2003). Hypotensive effect of butein via the inhibition of angiotensin converting enzyme. Biol. Pharm. Bull..

[93] Liu J.-C., Hsu F.-L., Tsai J.-C., Chan P., Liu J.Y.-H., Thomas G.N., Tomlinson B., Lo M.-Y., Lin J.-Y. (2003). Antihypertensive effects of tannins isolated from traditional chinese herbs as non-specific inhibitors of angiontensin converting enzyme. Life Sci..

[94] Zhao X., Gu Z., Attele A.S., Yuan C.-S. (1999). Effects of quercetin on the release of endothelin, prostacyclin and tissue plasminogen activator from human endothelial cells in culture. J. Ethnopharmacol..

[95] Negrão R., Costa R., Duarte D., Gomes T.T., Azevedo I., Soares R. (2013). Different effects of catechin on angiogenesis and inflammation depending on VEGF levels. J. Nutr. Biochem..

[96] Negrao R., Duarte D., Costa R., Soares R. (2013). Isoxanthohumol modulates angiogenesis and inflammation via vascular endothelial growth factor receptor, tumor necrosis factor α and nuclear factor κB pathways. Biofactors.

[97] Kenny T.P., Keen C.L., Jones P., Kung H.J., Schmitz H.H., Gershwin M.E. (2004). Cocoa procyanidins inhibit proliferation and angiogenic signals in human dermal microvascular endothelial cells following stimulation by low-level H_2_O_2_. Exp. Biol. Med..

[98] Scoditti E., Calabriso N., Massaro M., Pellegrino M., Storelli C., Martines G., De Caterina R., Carluccio M.A. (2012). Mediterranean diet polyphenols reduce inflammatory angiogenesis through MMP-9 and COX-2 inhibition in human vascular endothelial cells: A potentially protective mechanism in atherosclerotic vascular disease and cancer. Arch. Biochem. Biophys..

[99] Oku N., Matsukawa M., Yamakawa S., Asai T., Yahara S., Hashimoto F., Akizawa T. (2003). Inhibitory effect of green tea polyphenols on membrane-type 1 matrix metalloproteinase, MT1-MMP. Biol. Pharm. Bull..

[100] Elgass S., Cooper A., Chopra M. (2012). Lycopene inhibits angiogenesis in human umbilical vein endothelial cells and rat aortic rings. Br. J. Nutr..

[101] Zhao M., Tang S.-N., Marsh J.L., Shankar S., Srivastava R.K. (2013). Ellagic acid inhibits human pancreatic cancer growth in BALB c nude mice. Cancer Lett..

[102] Kim J.J., Tan Y., Xiao L., Sun Y.L., Qu X. (2013). Green tea polyphenol epigallocatechin-3-gallate enhance glycogen synthesis and inhibit lipogenesis in hepatocytes. Biomed Res. Int..

[103] Collins Q.F., Liu H.Y., Pi J., Liu Z., Quon M.J., Cao W. (2007). Epigallocatechin-3-gallate (EGCG), a green tea polyphenol, suppresses hepatic gluconeogenesis through 5′-AMP-activated protein kinase. J. Biol. Chem..

[104] Hwang P.Y., Gyun Kim H., Choi J.H., Truong Do M., Tran T.P., Chun H.K., Chung Y.C., Jeong T.C., Jeong H.G. (2013). 3-Caffeoyl, 4-dihydrocaffeoylquinic acid from Salicornia herbacea attenuates high glucose-induced hepatic lipogenesis in human HepG2 cells through activation of the liver kinase B1 and silent information regulator T1/APMK-dependent pathway. Mol. Nutr. Food Res..

[105] Liu Y.X., Wang D., Zhang D., Lv Y.C., Wei Y., Wu W., Zhou F., Tang M.M., Mao T., Li M.M. (2011). Inhibitory effect of blueberry polyphenolic compounds on oleic acid-induced hepatic steatosis *in vitro*. J. Agric. Food Chem..

[106] Wang H.-C., Chung P.-J., Wu C.-H., Lan K.-P., Yang M.-Y., Wang C.-J. (2011). *Solanum nigrum* L. Polyphenolic extract inhibits hepatocarcinoma cell growth by inducing G2/M phase arrest and apoptosis. J. Sci. Food Agric..

[107] Wu C.-H., Ou T.-T., Chang C.-H., Chang X.-Z., Yang M.-Y., Wang C.-J. (2013). The polyphenol extract from sechium edule shoots inhibits lipogenesis and stimulates lipolysis via activation of AMPK signals in HepG2 cells. J. Agric. Food Chem..

[108] Choi Y.J., Suh H.R., Yoon Y., Lee K.J., Kim D.G., Kim S., Lee B.H. (2014). Protective effect of resveratrol derivatives on high-fat diet induced fatty liver by activating AMP-activated protein kinase. Arch. Pharmacal Res..

[109] Kang O.H., Kim S.B., Seo Y.S., Joung D.K., Mun S.H., Choi J.G., Lee Y.M., Kang D.G., Lee H.S., Kwon D.Y. (2013). Curcumin decreases oleic acid-induced lipid accumulation via AMPK phosphorylation in hepatocarcinoma cells. Eur. Rev. Med. Pharmacol. Sci..

[110] Gnoni G.V., Paglialonga G. (2009). Resveratrol inhibits fatty acid and triacylglycerol synthesis in rat hepatocytes. Eur. J. Clin. Investig..

[111] Zang M., Xu S., Maitland-Toolan K.A., Zuccollo A., Hou X., Jiang B., Wierzbicki M., Verbeuren T.J., Cohen R.A. (2006). Polyphenols stimulate AMP-activated protein kinase, lower lipids, and inhibit accelerated atherosclerosis in diabetic LDL receptor-deficient mice. Diabetes.

[112] Shang J., Chen L.L., Xiao F.X., Sun H., Ding H.C., Xiao H. (2008). Resveratrol improves non-alcoholic fatty liver disease by activating AMP-activated protein kinase. Acta Pharmacol. Sin..

[113] Wang G.L., Fu Y.C., Xu W.C., Feng Y.Q., Fang S.R., Zhou X.H. (2009). Resveratrol inhibits the expression of SREBP1 in cell model of steatosis via Sirt1-FOXO1 signaling pathway. Biochem. Biophys. Res. Commun..

[114] Vidyashankar S., Sandeep Varma R., Patki P.S. (2013). Quercetin ameliorate insulin resistance and up-regulates cellular antioxidants during oleic acid induced hepatic steatosis in HepG2 cells. Toxicol. Vitro.

[115] Guo H., Li D., Ling W., Feng X., Xia M. (2011). Anthocyanin inhibits high glucose-induced hepatic mtGPAT1 activation and prevents fatty acid synthesis through PKCζ. J. Lipid Res..

[116] Wang Y. (2012). Small lipid-binding proteins in regulating endothelial and vascular functions: Focusing on adipocyte fatty acid binding protein and lipocalin-2. Br. J. Pharmacol..

[117] Guo H., Liu G., Zhong R., Wang Y., Wang D., Xia M. (2012). Cyanidin-3-*O*-β-glucoside regulates fatty acid metabolism via an AMP-activated protein kinase-dependent signaling pathway in human HepG2 cells. Lipids Health Dis..

[118] Zhu W., Jia Q., Wang Y., Zhang Y., Xia M. (2012). The anthocyanin cyanidin-3-*O*-β-glucoside, a flavonoid, increases hepatic glutathione synthesis and protects hepatocytes against reactive oxygen species during hyperglycemia: Involvement of a cAMP-PKA-dependent signaling pathway. Free Radic. Biol. Med..

[119] Hwang Y.P., Choi J.H., Han E.H., Kim H.G., Wee J.H., Jung K.O., Jung K.H., Kwon K.I., Jeong T.C., Chung Y.C. (2011). Purple sweet potato anthocyanins attenuate hepatic lipid accumulation through activating adenosine monophosphate-activated protein kinase in human HepG2 cells and obese mice. Nutr. Res..

[120] Chang J.J., Hsu M.J., Huang H.P., Chung D.J., Chang Y.C., Wang C.J. (2013). Mulberry anthocyanins inhibit oleic acid induced lipid accumulation by reduction of lipogenesis and promotion of hepatic lipid clearance. J. Agric. Food Chem..

[121] Cordero-Herrera I., Martin M.A., Goya L., Ramos S. (2014). Cocoa flavonoids attenuate high glucose-induced insulin signalling blockade and modulate glucose uptake and production in human HepG2 cells. Food Chem. Toxicol..

[122] Baselga-Escudero L., Blade C., Ribas-Latre A., Casanova E., Salvado M.J., Arola L., Arola-Arnal A. (2012). Grape seed proanthocyanidins repress the hepatic lipid regulators miR-33 and miR-122 in rats. Mol. Nutr. Food Res..

[123] Cho B.O., Ryu H.W., Jin C.H., Choi D.S., Kang S.Y., Kim D.S., Byun M.W., Jeong I.Y. (2011). Blackberry extract attenuates oxidative stress through up-regulation of Nrf2-dependent antioxidant enzymes in carbon tetrachloride-treated rats. J. Agric. Food Chem..

[124] Jia Y.Y., Kim J.Y., Jun H.J., Kim S.J., Lee J.H., Hoang M.H., Kim H.S., Chang H.I., Hwang K.Y., Um S.J. (2013). Cyanidin is an agonistic ligand for peroxisome proliferator-activated receptor-α reducing hepatic lipid. BBA-Mol. Cell Biol. Lett..

[125] Bursill C.A., Roach P.D. (2006). Modulation of cholesterol metabolism by the green tea polyphenol (−)-epigallocatechin gallate in cultured human liver (HepG2) cells. J. Agric. Food Chem..

[126] Granado-Serrano A.B., Martín M.A., Bravo L., Goya L., Ramos S. (2006). Quercetin induces apoptosis via caspase activation, regulation of Bcl-2, and inhibition of PI-3-kinase/Akt and Erk pathways in a human hepatoma cell line (HepG2). J. Nutr..

[127] Granado-Serrano A.B., Martin M.A., Izquierdo-Pulido M., Goya L., Bravo L., Ramos S. (2007). Molecular mechanisms of (-)-epicatechin and chlorogenic acid on the regulation of the apoptotic and survival/proliferation pathways in a human hepatoma cell line. J. Agric. Food Chem..

[128] Pal S., Ho N., Santos C., Dubois P., Mamo J., Croft K., Allister E. (2003). Red wine polyphenolics increase ldl receptor expression and activity and suppress the secretion of ApoB100 from human HepG2 cells. J. Nutr..

[129] Nakagawa S., Kojima Y., Sekino K., Yamato S. (2013). Effect of polyphenols on 3-hydroxy-3-methylglutaryl-coenzyme a lyase activity in human hepatoma HepG2 cell extracts. Biol. Pharm. Bull..

[130] Monga J., Pandit S., Chauhan R.S., Chauhan C.S., Chauhan S.S., Sharma M. (2013). Growth inhibition and apoptosis induction by (+)-cyanidan-3-ol in hepatocellular carcinoma. PLoS ONE.

[131] Nishikawa T., Nakajima T., Moriguchi M., Jo M., Sekoguchi S., Ishii M., Takashima H., Katagishi T., Kimura H., Minami M. (2006). A green tea polyphenol, epigalocatechin-3-gallate, induces apoptosis of human hepatocellular carcinoma, possibly through inhibition of Bcl-2 family proteins. J. Hepatol..

[132] Ou X., Chen Y., Cheng X., Zhang X., He Q. (2014). Potentiation of resveratrol-induced apoptosis by matrine in human hepatoma HepG2 cells. Oncol. Rep..

[133] Murugan R.S., Priyadarsini R.V., Ramalingam K., Hara Y., Karunagaran D., Nagini S. (2010). Intrinsic apoptosis and NF-κB signaling are potential molecular targets for chemoprevention by black tea polyphenols in HepG2 cells *in vitro* and in a rat hepatocarcinogenesis model *in vivo*. Food Chem. Toxicol..

[134] Ramiro E., Franch A., Castellote C., Perez-Cano F., Permanyer J., Izquierdo-Pulido M., Castell M. (2005). Flavonoids from theobroma cacao down-regulate inflammatory mediators. J. Agric. Food Chem..

[135] Cullen J.P., Morrow D., Jin Y., von Offenberg Sweeney N., Sitzmann J.V., Cahill P.A., Redmond E.M. (2007). Resveratrol inhibits expression and binding activity of the monocyte chemotactic protein-1 receptor, CCR2, on THP-1 monocytes. Atherosclerosis.

[136] Wang Q., Xia M., Liu C., Guo H., Ye Q., Hu Y., Zhang Y., Hou M., Zhu H., Ma J. (2008). Cyanidin-3-*O*-β-glucoside inhibits iNOS and COX-2 expression by inducing liver x receptor α activation in THP-1 macrophages. Life Sci..

[137] Chacon M.R., Ceperuelo-Mallafre V., Maymo-Masip E., Mateo-Sanz J.M., Arola L., Guitierrez C., Fernandez-Real J.M., Ardevol A., Simon I., Vendrell J. (2009). Grape-seed procyanidins modulate inflammation on human differentiated adipocytes *in vitro*. Cytokine.

[138] Kang O.H., Jang H.J., Chae H.S., Oh Y.C., Choi J.G., Lee Y.S., Kim J.H., Kim Y.C., Sohn D.H., Park H. (2009). Anti-inflammatory mechanisms of resveratrol in activated HMC-1 cells: Pivotal roles of NF-κ B and MAPK. Pharmacol. Res..

[139] Huang N., Hauck C., Yum M.Y., Rizshsky L., Widrlechner M.P., Mccoy J.A., Murphy P.A., Dixon P.M., Nikolau B.J., Birt D.F. (2009). Rosmarinic acid in prunella vulgaris ethanol extract inhibits lipopolysaccharide-induced prostaglandin E2 and nitric oxide in RAW 264.7 mouse macrophages. J. Agric. Food Chem..

[140] Zhang X.M., Cao J., Zhong L.F. (2009). Hydroxytyrosol inhibits pro-inflammatory cytokines, iNOS, and COX-2 expression in human monocytic cells. Naunyn-Schmiedeberg’s Arch. Pharmacology.

[141] Dell’Agli M., Fagnani R., Galli G.V., Maschi O., Gilardi F., Bellosta S., Crestani M., Bosisio E., De Fabiani E., Caruso D. (2010). Olive oil phenols modulate the expression of metalloproteinase 9 in THP-1 cells by acting on nuclear factor-κB signaling. J. Agric. Food Chem..

[142] dos Santos M.D., Chen G.J., Almeida M.C., Soares D.M., de Souza G.E.P., Lopes N.P., Lantz R.C. (2010). Effects of caffeoylquinic acid derivatives and C-flavonoid from lychnophora ericoides on *in vitro* inflammatory mediator production. Nat. Prod. Commun..

[143] Kuppan G., Balasubramanyam J., Monickaraj F., Srinivasan G., Mohan V., Balasubramanyam M. (2010). Transcriptional regulation of cytokines and oxidative stress by gallic acid in human THP-1 monocytes. Cytokine.

[144] Zhang Y., Lian F., Zhu Y., Xia M., Wang Q., Ling W., Wang X.D. (2010). Cyanidin-3-*O*-β-glucoside inhibits lps-induced expression of inflammatory mediators through decreasing IκBα phosphorylation in THP-1 cells. Inflamm. Res..

[145] Wu C.H., Huang H.W., Lin J.A., Huang S.M., Yen G.C. (2011). The proglycation effect of caffeic acid leads to the elevation of oxidative stress and inflammation in monocytes, macrophages and vascular endothelial cells. J. Nutr. Biochem..

[146] Yun J.M., Jialal I., Devaraj S. (2011). Epigenetic regulation of high glucose-induced proinflammatory cytokine production in monocytes by curcumin. J. Nutr. Biochem..

[147] Essafi-Benkhadir K., Refai A., Riahi I., Fattouch S., Karoui H., Essafi M. (2012). Quince (*Cydonia oblonga* miller) peel polyphenols modulates LPS-induced inflammation in human THP-1-derived macrophages through NF-kB, p38 MAPK and Akt inhibition. Biochem. Biophys. Res. Commun..

[148] Peterson L.W., Artis D. (2014). Intestinal epithelial cells: Regulators of barrier function and immune homeostasis. Nat. Rev. Immunol..

[149] Pinto M., Robine-Leon S., Appay M.D., Kedinger M., Triadou N., Dussaulx E., Lacroix B., Simon-Assman P., Haffen K., Fogh J. (1983). Enterocyte-like differentiation and polarization of the human colon carcinoma cell line Caco-2 in culture. Biol. Cell.

[150] Hidalgo I.J., Raub T.J., Borchardt R.T. (1989). Characterization of the human colon carcinoma cell line (Caco-2) as a model system for intestinal epithelial permeability. Gastroenterology.

[151] Neutra M., Louvard D. (1989). Differentiation of intestinal cells *in vitro*. Mod. Cell Biol..

[152] Mircheff A.K., Wright E.M. (1976). Analytical isolation of plasma membranes of intestinal epithelial cells: Identification of Na, K-ATPase rich membranes and the distribution of the enzyme activities. J. Membr. Biol..

[153] Zweibaum A., Pinto M., Chevalier G., Dussaulx E., Triadou N., Lacroix B., Haffen K., Brun J.L., Rousset M. (1985). Enterocytic differentiation of a subpopulation of the human colon tumor cell line HT-29 selected for growth in sugar-free medium and its inhibition by glucose. J. Cell Physiol..

[154] Gonzales G., Van Camp J., Zotti M., Kobayashi V., Grootaert C., Raes K., Smagghe G. (2014). Two- and three-dimensional quantitative structure–permeability relationship of flavonoids in Caco-2 cells using stepwise multiple linear regression (SMLR), partial least squares regression (PLSR), and pharmacophore (GALAHAD)-based comparative molecular similarity index analysis (COMSIA). Med. Chem. Res..

[155] Hers I., Tavare J.M. (2005). Mechanism of feedback regulation of insulin receptor substrate-1 phosphorylation in primary adipocytes. Biochem. J..

[156] Armoni M., Harel C., Karnieli E. (2007). Transcriptional regulation of the GLUT4 gene: From PPAR-γ and FOXO1 to FFA and inflammation. Trends Endocrinol. Metab..

[157] Hui X., Lam K.S., Vanhoutte P.M., Xu A. (2012). Adiponectin and cardiovascular health: An update. Br. J. Pharmacol..

[158] Li F.Y.L., Cheng K.K.Y., Lam K.S.L., Vanhoutte P.M., Xu A. (2011). Cross-talk between adipose tissue and vasculature: Role of adiponectin. Acta Physiol..

[159] Robertson S.A., Rae C.J., Graham A. (2009). Induction of angiogenesis by murine resistin: Putative role of PI3-kinase and no-dependent pathways. Regul. Pept..

[160] Kunduzova O., Alet N., Delesque-Touchard N., Millet L., Castan-Laurell I., Muller C., Dray C., Schaeffer P., Herault J.P., Savi P. (2008). Apelin/APJ signaling system: A potential link between adipose tissue and endothelial angiogenic processes. FASEB J..

[161] Kalea A.Z., Batlle D. (2010). Apelin and ACE2 in cardiovascular disease. Curr. Opin. Investig. Drugs.

[162] Heilbronn L.K., Campbell L.V. (2008). Adipose tissue macrophages, low grade inflammation and insulin resistance in human obesity. Curr. Pharm. Des..

[163] Wabitsch M., Brenner R.E., Melzner I., Braun M., Moller P., Heinze E., Debatin K.M., Hauner H. (2001). Characterization of a human preadipocyte cell strain with high capacity for adipose differentiation. Int. J. Obes. Relat. Metab. Disord..

[164] Zilberfarb V., Siquier K., Strosberg A.D., Issad T. (2001). Effect of dexamethasone on adipocyte differentiation markers and tumour necrosis factor-α expression in human PAZ6 cells. Diabetologia.

[165] Forest C., Czerucka D., Negrel R., Ailhaud G. (1983). Establishment of a human cell line after transformation by a plasmid containing the early region of the SV40 genome. Cell Biol. Int. Rep..

[166] Tontonoz P., Singer S., Forman B.M., Sarraf P., Fletcher J.A., Fletcher C.D., Brun R.P., Mueller E., Altiok S., Oppenheim H. (1997). Terminal differentiation of human liposarcoma cells induced by ligands for peroxisome proliferator-activated receptor γ and the retinoid X receptor. Proc. Natl. Acad. Sci. USA.

[167] Hugo E.R., Brandebourg T.D., Comstock C.E.S., Gersin K.S., Sussman J.J., Ben-Jonathan N. (2006). LS14: A novel human adipocyte cell line that produces prolactin. Endocrinology.

[168] Yamagata K., Tagami M., Yamori Y. (2015). Dietary polyphenols regulate endothelial function and prevent cardiovascular disease. Nutrition.

[169] Targosz-Korecka M., Brzezinka G.D., Malek K.E., Stepien E., Szymonski M. (2013). Stiffness memory of EA.Hy926 endothelial cells in response to chronic hyperglycemia. Cardiovasc. Diabetol..

[170] Ades E.W., Candal F.J., Swerlick R.A., George V.G., Summers S., Bosse D.C., Lawley T.J. (1992). HMEC-1: Establishment of an immortalized human microvascular endothelial cell line. J. Investig. Dermatol..

[171] Masuzawa M., Fujimura T., Hamada Y., Fujita Y., Hara H., Nishiyama S., Katsuoka K., Tamauchi H., Sakurai Y. (1999). Establishment of a human hemangiosarcoma cell line (ISO-HAS). Int. J. Cancer.

[172] Ma X., Sickmann A., Pietsch J., Wildgruber R., Weber G., Infanger M., Bauer J., Grimm D. (2014). Proteomic differences between microvascular endothelial cells and the EA.Hy926 cell line forming three-dimensional structures. Proteomics.

[173] Aguirre L., Portillo M.P., Hijona E., Bujanda L. (2014). Effects of resveratrol and other polyphenols in hepatic steatosis. World J. Gastroenterol..

[174] Walldius G. (2010). Apolipoprotein B (apoB) more closely related to subclinical atherosclerosis than non-HDL cholesterol and LDL cholesterol. J. Internal Med..

[175] Wilkening S., Stahl F., Bader A. (2003). Comparison of primary human hepatocytes and hepatoma cell line HepG2 with regard to their biotransformation properties. Drug Metab. Dispos..

[176] Bokhari M., Carnachan R.J., Cameron N.R., Przyborski S.A. (2007). Culture of HepG2 liver cells on three dimensional polystyrene scaffolds enhances cell structure and function during toxicological challenge. J. Anat..

[177] Khanduja K.L., Avti P.K., Kumar S., Mittal N., Sohi K.K., Pathak C.M. (2006). Anti-apoptotic activity of caffeic acid, ellagic acid and ferulic acid in normal human peripheral blood mononuclear cells: A Bcl-2 independent mechanism. Biochim. Biophys. Acta.

[178] Mir M.A., Agrewala J.N. (2008). Dietary polyphenols in modulation of the immune system. Nova Sci. Publ..

[179] Frostegard J. (2013). Immunity, atherosclerosis and cardiovascular disease. BMC Med..

[180] Auwerx J. (1991). The human leukemia cell line, THP-1: A multifacetted model for the study of monocyte-macrophage differentiation. Experientia.

[181] Sak K. (2014). Cytotoxicity of dietary flavonoids on different human cancer types. Pharmacogn. Rev..

[182] Harrison D.G., Gongora M.C. (2009). Oxidative stress and hypertension. Med. Clin. N. Am..

[183] Ou K.L., Hosseinkhani H. (2014). Development of 3D *in vitro* technology for medical applications. Int. J. Mol. Sci..

[184] Guri A., Gulseren I., Corredig M. (2013). Utilization of solid lipid nanoparticles for enhanced delivery of curcumin in cocultures of HT29-MTX and Caco-2 cells. Food Funct..

[185] Le Dréan G., Haure-Mirande V., Ferrier L., Bonnet C., Hulin P., de Coppet P., Segain J.-P. (2013). Visceral adipose tissue and leptin increase colonic epithelial tight junction permeability via a RhoA-ROCK-dependent pathway. FASEB J..

[186] Maaser C., Schoeppner S., Kucharzik T., Kraft M., Schoenherr E., Domschke W., Luegering N. (2001). Colonic epithelial cells induce endothelial cell expression of ICAM-1 and VCAM-1 by a NF-κB-dependent mechanism. Clin. Exp. Immunol..

[187] Zgouras D., Wachtershauser A., Frings D., Stein J. (2003). Butyrate impairs intestinal tumor cell-induced angiogenesis by inhibiting HIF-1α nuclear translocation. Biochem. Biophys. Res. Commun..

[188] Kuntz S., Asseburg H., Dold S., Rompp A., Frohling B., Kunz C., Rudloff S. (2015). Inhibition of low-grade inflammation by anthocyanins from grape extract in an *in vitro* epithelial-endothelial co-culture model. Food Funct..

[189] Woitiski C.B., Sarmento B., Carvalho R.A., Neufeld R.J., Veiga F. (2011). Facilitated nanoscale delivery of insulin across intestinal membrane models. Int. J. Pharm..

[190] Nollevaux G., Deville C., El Moualij B., Zorzi W., Deloyer P., Schneider Y.J., Peulen O., Dandrifosse G. (2006). Development of a serum-free co-culture of human intestinal epithelium cell-lines (Caco-2/HT29–5M21). BMC Cell Biol..

[191] Laparra J.M., Glahn R.P., Miller D.D. (2009). Different responses of fe transporters in Caco-2/HT29-MTX cocultures than in independent Caco-2 cell cultures. Cell Biol. Int..

[192] Yao L., Friel J.K., Suh M., Diehl-Jones W.L. (2010). Antioxidant properties of breast milk in a novel *in vitro* digestion/enterocyte model. J. Pediatr. Gastroenterol. Nutr..

[193] Bouwmeester H., Poortman J., Peters R.J., Wijma E., Kramer E., Makama S., Puspitaninganindita K., Marvin H.J.P., Peijnenburg A.A.C.M., Hendriksen P.J.M. (2011). Characterization of translocation of silver nanoparticles and effects on whole-genome gene expression using an *in vitro* intestinal epithelium coculture model. ACS Nano.

[194] Ouattara D.A., Choi S.-H., Sakai Y., Péry A.R.R., Brochot C. (2011). Kinetic modelling of *in vitro* cell-based assays to characterize non-specific bindings and ADME processes in a static and a perfused fluidic system. Toxicol. Lett..

[195] Sakai Y., Fukuda O., Choi S.H., Sakoda A. (2003). Development of a biohybrid simulator for absorption and biotransformation processes in humans based on *in vitro* models of small intestine and liver tissues. J. Artif. Organs.

[196] Rossi C., Guantario B., Ferruzza S., Guguen-Guillouzo C., Sambuy Y., Scarino M.L., Bellovino D. (2012). Co-cultures of enterocytes and hepatocytes for retinoid transport and metabolism. Toxicol. Vitro.

[197] Satsu H., Yokoyama T., Ogawa N., Fujiwara-Hatano Y., Shimizu M. (2001). The changes in the neuronal PC12 and the intestinal epithelial Caco-2 cells during the coculture. The functional analysis using an *in vitro* coculture system. Cytotechnology.

[198] Xiao W.-D., Chen W., Sun L.-H., Wang W.-S., Zhou S.-W., Yang H. (2011). The protective effect of enteric glial cells on intestinal epithelial barrier function is enhanced by inhibiting inducible nitric oxide synthase activity under lipopolysaccharide stimulation. Mol. Cell. Neurosci..

[199] Flamant M., Aubert P., Rolli-Derkinderen M., Bourreille A., Neunlist M.R., Mahé M.M., Meurette G., Marteyn B., Savidge T., Galmiche J.P. (2011). Enteric glia protect against shigella flexneri invasion in intestinal epithelial cells: A role for S-nitrosoglutathione. Gut.

[200] Holland-Cunz S., Bainczyk S., Hagl C., Wink E., Wedel T., Back W., Schafer K.H. (2004). Three-dimensional co-culture model of enterocytes and primary enteric neuronal tissue. Pediatr. Surg. Int..

[201] Townley A.K., Schmidt K., Hodgson L., Stephens D.J. (2012). Epithelial organization and cyst lumen expansion require efficient Sec13–Sec31-driven secretion. J. Cell Sci..

[202] Lahar N., Lei N.Y., Wang J., Jabaji Z., Tung S.C., Joshi V., Lewis M., Stelzner M., Martin M.G., Dunn J.C. (2011). Intestinal subepithelial myofibroblasts support *in vitro* and *in vivo* growth of human small intestinal epithelium. PLoS ONE.

[203] Yoshikawa T., Hamada S., Otsuji E., Tsujimoto H., Hagiwara A. (2011). Endocrine differentiation of rat enterocytes in long-term three-dimensional co-culture with intestinal myofibroblasts. Vitro Cell. Dev. Biol. Anim..

[204] Viney M.E., Bullock A.J., Day M.J., MacNeil S. (2009). Co-culture of intestinal epithelial and stromal cells in 3D collagen-based environments. Regen. Med..

[205] Schmohl M., Schneiderhan-Marra N., Baur N., Hefner K., Blum M., Stein G.M., Joos T.O., Schmolz M. (2012). Characterization of immunologically active drugs in a novel organotypic co-culture model of the human gut and whole blood. Int. Immunopharmacol..

[206] Pozo-Rubio T., Mujico J.R., Marcos A., Puertollano E., Nadal I., Sanz Y., Nova E. (2011). Immunostimulatory effect of faecal Bifidobacterium species of breast-fed and formula-fed infants in a peripheral blood mononuclear cell/Caco-2 co-culture system. Br. J. Nutr..

[207] Tiscornia I., Sanchez-Martins V., Hernandez A., Bollati-Fogolin M. (2012). Human monocyte-derived dendritic cells from leukoreduction system chambers after plateletpheresis are functional in an *in vitro* co-culture assay with intestinal epithelial cells. J. Immunol. Methods.

[208] Zoumpopoulou G., Tsakalidou E., Dewulf J., Pot B., Grangette C. (2009). Differential crosstalk between epithelial cells, dendritic cells and bacteria in a co-culture model. Int. J. Food Microbiol..

[209] Rimoldi M., Chieppa M., Larghi P., Vulcano M., Allavena P., Rescigno M. (2005). Monocyte-derived dendritic cells activated by bacteria or by bacteria-stimulated epithelial cells are functionally different. Blood.

[210] Le Hegarat L., Huet S., Fessard V. (2012). A co-culture system of human intestinal Caco-2 cells and lymphoblastoid TK6 cells for investigating the genotoxicity of oral compounds. Mutagenesis.

[211] Ishimoto Y., Satsu H., Totsuka M., Shimizu M. (2011). Iex-1 suppresses apoptotic damage in human intestinal epithelial Caco-2 cells induced by co-culturing with macrophage-like THP-1 cells. Biosci. Rep..

[212] Tanoue T., Nishitani Y., Kanazawa K., Hashimoto T., Mizuno M. (2008). *In vitro* model to estimate gut inflammation using co-cultured Caco-2 and RAW264.7 cells. Biochem. Biophys. Res. Commun..

[213] Chen J., Ng C.P., Tsang L.L., Ho L.S., Xu P.H., Rowlands D.K., Gao J.Y., Chung Y.W., Li T.Y., Chan H.C. (2009). Altered expression of inflammatory cytokine receptors in response to LPS challenge through interaction between intestinal epithelial cells and lymphocytes of Peyer’s patch. Cell Biol. Int..

[214] Stybayeva G., Zhu H., Ramanculov E., Dandekar S., George M., Revzin A. (2009). Micropatterned co-cultures of T-lymphocytes and epithelial cells as a model of mucosal immune system. Biochem. Biophys. Res. Commun..

[215] Thierry A.C., Bernasconi E., Mercenier A., Corthésy B. (2009). Conditioned polarized Caco-2 cell monolayers allow to discriminate for the ability of gut-derived microorganisms to modulate permeability and antigen-induced basophil degranulation. Clin. Exp. Allergy.

[216] Wilcz-Villega E.M., McClean S., O’Sullivan M.A. (2013). Mast cell tryptase reduces junctional adhesion molecule-A (JAM-A) expression in intestinal epithelial cells: Implications for the mechanisms of barrier dysfunction in irritable bowel syndrome. Am. J. Gastroenterol..

[217] Antunes F., Andrade F., Araújo F., Ferreira D., Sarmento B. (2013). Establishment of a triple co-culture *in vitro* cell models to study intestinal absorption of peptide drugs. Eur. J. Pharm. Biopharm..

[218] Li N., Wang D., Sui Z., Qi X., Ji L., Wang X., Yang L. (2013). Development of an improved three-dimensional *in vitro* intestinal mucosa model for drug absorption evaluation. Tissue Eng..

[219] Leonard F., Ali H., Collnot E.M., Crielaard B.J., Lammers T., Storm G., Lehr C.M. (2012). Screening of budesonide nanoformulations for treatment of inflammatory bowel disease in an inflamed 3D cell-culture model. Altex.

[220] Pusch J., Votteler M., Göhler S., Engl J., Hampel M., Walles H., Schenke-Layland K. (2011). The physiological performance of a three-dimensional model that mimics the microenvironment of the small intestine. Biomaterials.

[221] Paprocka M., Duś D., Mitterrand M., Lamerant-Fayel N., Kieda C. (2008). Flow cytometric assay for quantitative and qualitative evaluation of adhesive interactions of tumor cells with endothelial cells. Microvasc. Res..

[222] Reyes-Farias M., Vasquez K., Ovalle-Marin A., Fuentes F., Parra C., Quitral V., Jimenez P., Garcia-Diaz D.F. (2015). Chilean native fruit extracts inhibit inflammation linked to the pathogenic interaction between adipocytes and macrophages. J. Med. Food.

[223] Sakurai T., Kitadate K., Nishioka H., Fujii H., Kizaki T., Kondoh Y., Izawa T., Ishida H., Radak Z., Ohno H. (2010). Oligomerized grape seed polyphenols attenuate inflammatory changes due to antioxidative properties in coculture of adipocytes and macrophages. J. Nutr. Biochem..

[224] Brown J.M., Hazen S.L. (2015). The gut microbial endocrine organ: Bacterially derived signals driving cardiometabolic diseases. Ann. Rev. Med..

[225] Ginter E., Simko V. (2014). Gut microorganisms and cardiovascular disease: Carnitine is the answer. Bratisl. Med. J..

[226] Tilg H., Moschen A.R. (2014). Microbiota and diabetes: An evolving relationship. Gut.

[227] Coconnier M.H., Bernet M.F., Kerneis S., Chauviere G., Fourniat J., Servin A.L. (1993). Inhibition of adhesion of enteroinvasive pathogens to human intestinal Caco-2 cells by *Lactobacillus acidophilus* strain LB decreases bacterial invasion. FEMS Microbiol. Lett..

[228] Morelli L., Garbagna N., Rizzello F., Zonenschain D., Grossi E. (2006). *In vivo* association to human colon of *Lactobacillus paracasei* B21060: Map from biopsies. Dig. Liver Dis..

[229] Schiffrin E.J., Brassart D., Servin A.L., Rochat F., Donnet-Hughes A. (1997). Immune modulation of blood leukocytes in humans by lactic acid bacteria: Criteria for strain selection. Am. J. Clin. Nutr..

[230] Elliott S.N., Buret A., McKnight W., Miller M.J., Wallace J.L. (1998). Bacteria rapidly colonize and modulate healing of gastric ulcers in rats. Am. J. Physiol..

[231] Marzorati M., Vanhoecke B., De Ryck T., Sadaghian Sadabad M., Pinheiro I., Possemiers S., Van den Abbeele P., Derycke L., Bracke M., Pieters J. (2014). The HMI module: A new tool to study the host-microbiota interaction in the human gastrointestinal tract *in vitro*. BMC Microbiol..

[232] Kim H.J., Ingber D.E. (2013). Gut-on-a-chip microenvironment induces human intestinal cells to undergo villus differentiation. Integr. Biol..

